# Molecular Simulations of Cotranslational Protein Folding: Fragment Stabilities, Folding Cooperativity, and Trapping in the Ribosome

**DOI:** 10.1371/journal.pcbi.0020098

**Published:** 2006-07-28

**Authors:** Adrian H Elcock

**Affiliations:** Department of Biochemistry, University of Iowa, Iowa City, Iowa, United States of America; Harvard University, United States of America

## Abstract

Although molecular simulation methods have yielded valuable insights into mechanistic aspects of protein refolding in vitro, they have up to now not been used to model the folding of proteins as they are actually synthesized by the ribosome. To address this issue, we report here simulation studies of three model proteins: chymotrypsin inhibitor 2 (CI2), barnase, and Semliki forest virus protein (SFVP), and directly compare their folding during ribosome-mediated synthesis with their refolding from random, denatured conformations. To calibrate the methodology, simulations are first compared with in vitro data on the folding stabilities of N-terminal fragments of CI2 and barnase; the simulations reproduce the fact that both the stability and thermal folding cooperativity increase as fragments increase in length. Coupled simulations of synthesis and folding for the same two proteins are then described, showing that both fold essentially post-translationally, with mechanisms effectively identical to those for refolding. In both cases, confinement of the nascent polypeptide chain within the ribosome tunnel does not appear to promote significant formation of native structure during synthesis; there are however clear indications that the formation of structure within the nascent chain is sensitive to location within the ribosome tunnel, being subject to both gain and loss as the chain lengthens. Interestingly, simulations in which CI2 is artificially stabilized show a pronounced tendency to become trapped within the tunnel in partially folded conformations: non-cooperative folding, therefore, appears in the simulations to exert a detrimental effect on the rate at which fully folded conformations are formed. Finally, simulations of the two-domain protease module of SFVP, which experimentally folds cotranslationally, indicate that for multi-domain proteins, ribosome-mediated folding may follow different pathways from those taken during refolding. Taken together, these studies provide a first step toward developing more realistic methods for simulating protein folding as it occurs in vivo.

## Synopsis

The question of how proteins fold into their three-dimensional native conformations continues to be a subject of considerable interest, in large part because misfolding or aggregation of proteins is associated with a number of important diseases. Most previous research has focused on how proteins refold from denatured conformations in vitro, and much of the experimentally observed behavior has proven to be explicable with molecular simulations performed on computers. Recently attention has begun to move toward understanding protein folding as it occurs in vivo, which development requires, among other things, consideration of potential interactions with chaperonins and non-specific crowding effects due to the high macromolecular concentrations encountered in physiological conditions. Also under increasing consideration experimentally is the possibility that proteins might begin to fold while being synthesized (i.e., cotranslational folding), and the purpose of this work is therefore to develop and apply a first molecular simulation strategy capable of modeling this process. The simulations thus described, while not free of assumptions and approximations, nevertheless provide some intriguing glimpses into how the process of protein folding might be modulated through coupling to synthesis within the large ribosomal subunit.

## Introduction

Understanding the factors that affect protein folding, misfolding, and aggregation remains a subject of intense academic and medical interest, in part because of the now clear link between these processes and a variety of common diseases [1]. Significant insights into the process of protein refolding in vitro have been obtained through both experimental and theoretical techniques [2–5], and the influence of theoretical work is reflected in the presence of expressions such as “energy landscape” [6] and “contact order” [7] in the folding lexicon. In fact, a number of aspects of folding as it occurs in vitro are now quite well understood, even though this understanding has yet to translate into the realm of allowing routine, accurate prediction of protein structure [8].

Protein folding in vivo is considerably more complicated than folding in vitro for a number of reasons [1]. First, a variety of chaperonins play prominent roles in preventing or reversing misfolding and aggregation [9–11]. Second, folding of proteins can be coupled directly to their synthesis in a process termed “cotranslational folding” [12–15]. Finally, both the kinetics and thermodynamics of protein folding, and the potential for aggregation, may be significantly affected by the highly crowded conditions encountered physiologically [16–18]. In recognition of some of these issues, a number of theoretical and simulation studies have begun to address the possible effects of chaperonins [19,20], confinement [19,21,22], and crowding [23–25] on the behavior of protein systems. In contrast, far less attention has been paid to the question of how the gradual, ribosome-mediated extension of a nascent protein chain might impact its folding behavior: the only previous simulation studies of which we are aware have considered the coupling of synthesis and folding for lattice-based protein models in the absence of the ribosome [26,27]; the second of these works provided the intriguing result that cotranslational folding could cause a protein to adopt a metastable conformation different from its native conformation.

This paper describes the first molecular simulations of the cotranslational folding of proteins progressively synthesized within a structurally detailed model of the ribosomal large subunit. The method borrows a simple Gō-type [28] folding model of a type that has been successfully used to model the refolding kinetics of a large number of proteins (e.g., [29–32]), and combines it with a new, straightforward computational protocol that models synthesis of a nascent chain by the gradual addition of amino acids at the ribosome's peptidyltransferase active site. Importantly, although the simplified nature of the model means that folding occurs on an artificially short timescale, care is taken to ensure that the simulated rates of folding and synthesis are realistically matched. The simulations allow a direct view of the way that protein folding behavior is likely to be affected by coupling to synthesis within the ribosome, and serve as a first step toward developing models that more faithfully represent protein folding as it occurs in vivo.

## Results

### Stabilities of Fragments of Chymotrypsin Inhibitor 2 and Barnase

Although a molecular simulation method can be programmed to model the coupled synthesis and folding of proteins relatively easily, the credibility of simulations performed with the method rests on their ability to reproduce appropriate experimental data. Since translation of proteins occurs starting at the N-terminus, one straightforward test that can be imagined is whether the simulation model successfully captures the folding behavior of N-terminal fragments of different lengths: in this regard, an important body of data is a series of studies conducted by the Fersht group characterizing the behavior of a series of fragments of the model proteins chymotrypsin inhibitor 2 (CI2) [33–35] and barnase [36,37] (reviewed in [38]). For both proteins it has been shown that fragments with C-terminal residues deleted are markedly reduced in stability relative to the full-length protein; our first task, therefore, is to investigate whether the simulation model can reproduce this behavior. This has been done in the following way. First, the single adjustable parameter of the model—the energy well depth (ɛ) assigned to all favorable residue–residue interactions—has been adjusted so that the experimental free energy of folding (ΔG°_fold_) of each full-length protein at 300 K is approximately reproduced. Then, the same optimized energy parameter has been used in simulations of smaller fragments of the same protein in order to *predict* their behavior (see Materials and Methods).

**Figure 1:**
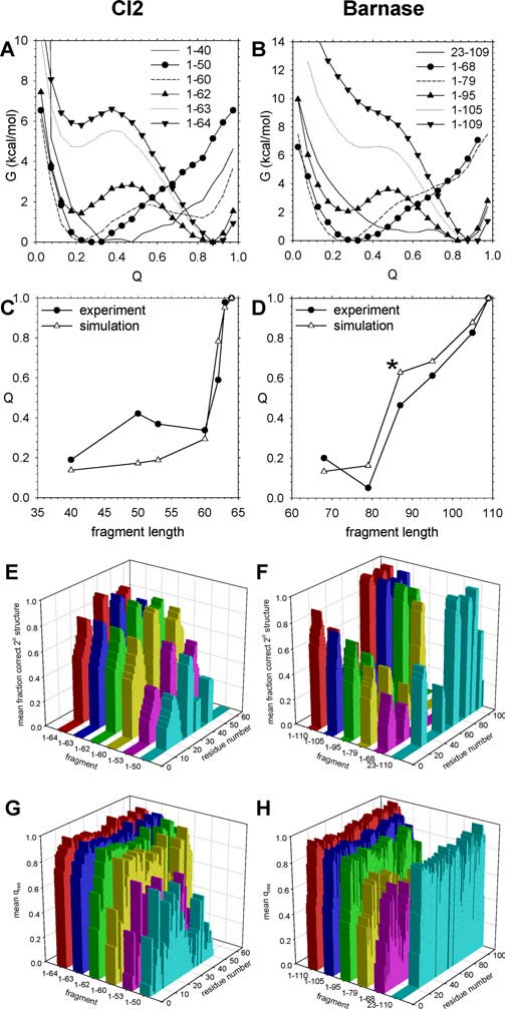
Folding Stabilities and Properties of CI2 and Barnase Fragments (A) Plot of free energy, G, versus Q at 300 K for six fragments of CI2. (B) Same as (A), but showing results for barnase. (C) Mean value of Q computed from simulations for CI2 compared with fluorescence intensities taken from Figure 10 of [34]. (D) Same as (C), but for barnase, and compared with fluorescence intensities taken from Table 1 of [36]. (E) Fractional population of correct secondary structure at all residues in CI2 fragments at 278 K. (F) Same as (E), but for barnase. (G) Mean values of Q_res_ at all residues in CI2 fragments at 278 K. (H) Same as (G), but for barnase.

Throughout this work we assume that the extent of folding in a given protein conformation can be adequately described in terms of the folding (reaction) coordinate “Q,” which is conventionally defined as the fraction of the set of native residue–residue contacts that are present in the conformation. Figure 1A shows the computed free energy (G) as a function of Q for full-length CI2 and its fragments at 300 K; Figure 1B shows the same information for fragments of barnase. For full-length CI2 (fragment 1–64), there are two clear minima on the one-dimensional energy landscape described by Q: a global minimum at Q ~ 0.9 that represents the folded state, and a local minimum at Q ~ 0.25 that represents the unfolded state. The free energy difference between the unfolded and folded state basins approximates the experimental folding free energy of −7 kcal/mol [39]; the energy parameter that produced these results was ɛ = 0.60 kcal/mol. For CI2 fragments that are close to full length (fragments 1–60, 1–62, and 1–63 in Figure 1A), the computed free energy landscapes also exhibit two minima, but there is a progressive decrease in the free energy difference between folded and unfolded states as the fragments become shorter. For the shortest CI2 fragments studied (1–40 and 1–50), only single, broad minima located at low Q values are obtained, indicating that both fragments are predominantly unfolded at 300 K. The results obtained for barnase are qualitatively similar (Figure 1B). In this case however, since for the full-length protein (fragment 1–109), a separate local minimum for the unfolded state is not completely resolved on the one-dimensional energy surface at 300 K (though it is at the folding temperature, T_fold_), it is less easy to match the experimental value (ΔG°_fold_ = −9 kcal/mol; [40]) with precision; the final chosen value of ɛ was 0.57 kcal/mol, which is very similar to that chosen for CI2. At no temperature does the one-dimensional free energy surface show the presence of the folding intermediate apparent in a previous Gō-model study of barnase refolding [29]. Again however, as with CI2, only those fragments that are close to full length (fragments 1–95 and 1–105) retain appreciable stability: there is a progressive loss of stability in the fragments of decreasing length (Figure 1B).

The one-dimensional free energy surfaces for fragments of CI2 and barnase show behavior that is for the most part qualitatively consistent with experiment, but there are more-quantitative comparisons that can be performed (compiled in the remaining panels of Figure 1). The most straightforward is to use the computed free energy profiles to calculate the mean value of Q adopted by each fragment at 300 K (Materials and Methods) and to compare these calculated values with a corresponding experimental measure of the fragments' conformational state. In the present case, the most convenient such measures are the fluorescence intensities reported by the Fersht group, which for both proteins have been argued to provide a reasonably direct measure of the extent of tertiary structure formation [34,36]. Comparisons of the computed mean Q values with these experimental fluorescence intensities are shown in Figure 1C for CI2 and Figure 1D for barnase. The agreement for both proteins is surprisingly good and particularly so in the case of barnase (Figure 1D), even when studies are extended to a C-terminal fragment (residues 23–109; asterisk in Figure 1D).

It is possible to conduct more-detailed analyses of the development of structure in the fragments. Figure 1E and 1G show the extent of secondary and tertiary structure formation, respectively, at individual residues in all studied fragments of CI2; note that for these purposes, we define tertiary structure in terms of a *residue-specific* folding reaction coordinate (Q_res_) for each residue (Materials and Methods), and note also that both secondary and tertiary structure are computed at 278 K to facilitate comparison with the Fersht group's measurements [34,35]. In terms of the computed secondary structure (Figure 1E), there is a low level of correct secondary structure formation in fragments 1–40 (unpublished data), 1–50, and 1–53, but this increases significantly between fragments 1–53 (magenta) and 1–60 (yellow). A similar trend is observed with the more tertiary structure–oriented measure Q_res_, (Figure 1G), indicating that in the simulations, the development of secondary and tertiary structure occurs in concert as the fragments increase in length. A concomitant development of secondary and tertiary structure is also observed experimentally, and an abrupt increase in both secondary structure and tertiary structure is also inferred; however, the experimental transition occurs considerably earlier, between fragments 1–50 and 1–53 [34,35]. Corresponding results for barnase are shown in Figure 1F and 1H. Again, secondary and tertiary structure develop largely in concert with each other in the simulations, but there appears to be a significant consolidation of tertiary structure between fragments 1–79 (yellow) and 1–95 (green; Figure 1H) that is not as readily apparent in the secondary structure plot (Figure 1F). Unfortunately, only limited nuclear magnetic resonance data could be obtained for fragment 1–95 because of aggregation issues [36]; experimentally, therefore, it can only be stated that there is a significant increase in tertiary structure somewhere between fragments 1–79 and 1–105, which is at least not inconsistent with the simulated behavior.

### Cooperativity of Thermal Unfolding for Fragments of CI2 and Barnase

**Figure 2:**
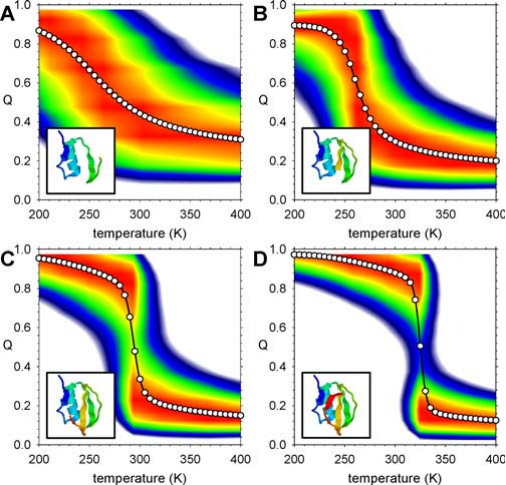
Folding Free Energy Surfaces for CI2 Fragments (A–D) Free energy surfaces for fragments comprising residues 1–40, 1–50, 1–60, and 1–64 (full length), respectively. Free energy (G) is shown on a continuous color scale from 0 (red) to +5 kcal/mol (white); symbols show the mean value of Q in 5-K intervals. Cartoons in bottom-left corner of each panel indicate the “native” structure of each fragment generated using RasMol [94].

Although the above comparisons indicate that the simple C_α_-based simulation model does not produce a perfect description of the folding behavior of fragments, they do indicate that it provides a good qualitative reproduction of the key experimental finding that substantial global structure formation in CI2 and barnase occurs only with fragments that are near to full length. A second important finding of the Fersht group's experimental studies of both CI2 and barnase is that the thermal unfolding of longer fragments is noticeably more cooperative than that of smaller fragments [34,36]. The question of whether this result is also reproduced by the simulation model has been answered by computing folding free energy surfaces over the range of temperatures from 200 K to 400 K; illustrations of some of the computed surfaces are shown for CI2 fragments 1–40, 1–50, 1–60, and 1–64 in Figure 2. As indicated by the mean value of the folding coordinate Q (open circles), increasing the temperature produces the expected effect of shifting the equilibrium from the folded state (Q ~ 0.9) to the unfolded state (Q ~ 0.2) for all fragments. Crucially, however, the thermal transition from the folded to the unfolded state becomes noticeably more abrupt (i.e., cooperative) as the fragments increase in length; a qualitatively identical result is also obtained with barnase fragments (Figure S1). The increase in the cooperativity of folding can be more clearly seen in plots of the proteins' heat capacities as a function of temperature (Figure S2): for both CI2 and barnase, the peak in the computed heat capacity that occurs at the transition midpoint becomes increasingly sharp as the fragments increase in length.

### Coupled Synthesis and Folding of CI2

**Figure 3:**
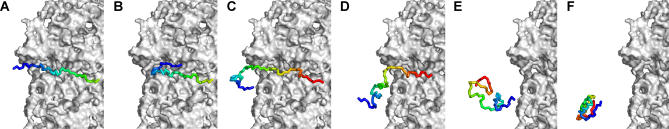
A Coupled Synthesis–Folding Simulation of CI2 Snapshots from a typical de novo folding simulation of CI2 in the presence of the ribosome (grey surface). The nascent chain is colored from blue (N-terminus) to red (C-terminus). Snapshots (A–F) are for timepoints 3.6 μs, 4 μs, 6 μs, 6.04 μs, 6.08 μs, and 6.23 μs, respectively; the length of the nascent protein in (A) and (B) is 40 and 44 residues, respectively, in all other panels, it is full length. This figure was prepared with PyMOL [95].

**Figure 4:**
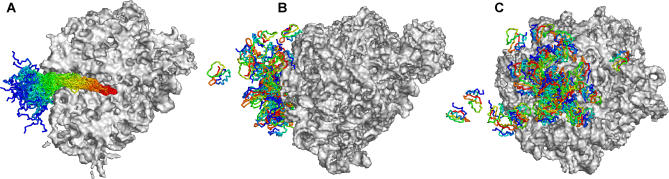
Fifty Coupled Synthesis–Folding Simulations of CI2 (A) Snapshots showing all 50 coupled synthesis–folding simulations of CI2 at the moment at which the final residue is released. (B) and (C) Snapshots for the same system at the moment that the native conformations are achieved; the two views are related by a 90° rotation around the vertical axis. Note that in this simulation model, the proteins do not diffuse far from the ribosome before completing folding.

The above results indicate that the simple simulation model used here qualitatively reproduces two key findings of the Fersht group's studies: (1) that a stable, folded protein does not form until the polypeptide begins to approach its full length, and (2) that the thermal cooperativity of the folding transition increases as the polypeptide reaches its full length. This provides confidence in extending the same basic approach to investigate how the folding behavior of proteins might be affected (or unaffected) by coupling to synthesis within the ribosome. To this end, a simple computational protocol was developed for modeling the progressive growth of a polypeptide chain within a structural model of the large ribosomal subunit (Materials and Methods). Simulation snapshots taken from a typical coupled synthesis–folding simulation of CI2 conducted with the same energy parameter used above (ɛ = 0.60 kcal/mol) are shown in Figure 3. As the nascent protein chain lengthens, the N-terminus gradually worms its way out of the ribosomal tunnel, first emerging into the solution environment when it is 35–40 residues long (Figure 3A); notably however, even after this point, it continues to make occasional returns toward the peptidyltransferase active site (Figure 3B), consistent with experimental cross-linking results reported for other proteins [41]. The protein's conformation at the moment that its final residue is added is shown in Figure 3C. After this point, matters proceed rapidly: the protein escapes from the ribosome and completes its “de novo” folding within approximately 230 ns, which, in the artificially rapid timescale of the simulations (see Discussion) is only 1/25th of the time taken to complete its synthesis. To obtain a reasonable sample of behavior, a total of 50 independent simulation trajectories were computed; snapshots of each trajectory taken at the moment of the nascent protein's release, and at the moment that the proteins complete de novo folding are superimposed in Figure 4.

**Figure 5:**
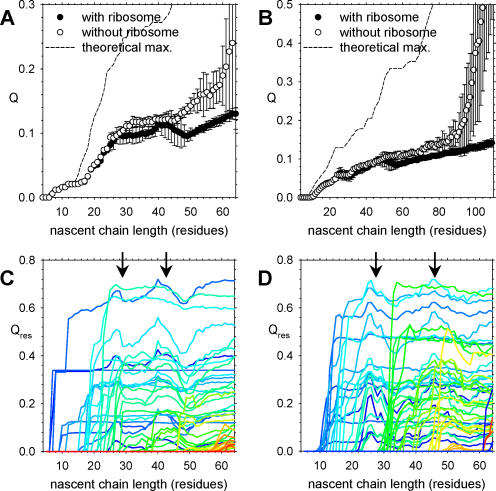
Effect of Confinement on Folding of CI2 and Barnase (A) Mean values of Q during coupled synthesis–folding of CI2 in the presence (filled circles) and absence (open circles) of the ribosome. (B) Same as (A), but for barnase. (C) Mean values of the residue-specific Q_res_ value during coupled synthesis–folding of CI2 in the presence of the ribosome; separate lines are plotted for each residue, colored from blue to red. Arrows indicate periods in which several of the Q_res_ values suddenly decrease in concert. (D) Same as (C), but for barnase; note that for clarity, only the period of synthesis of the first 60 residues is shown.

In the particular CI2 simulation illustrated in Figure 3, it is apparent that major elements of tertiary structure do not form in the protein until it is fully synthesized; this turns out to be true in all 50 simulations performed of the same system. Plots of the mean folding coordinate Q as a function of the nascent chain length for the simulations are shown for the period during synthesis as filled circles in Figure 5A; consistent with the low stabilities of N-terminal fragments shown earlier, the mean Q exhibited in these simulations is always much lower than the theoretical maximum (dashed line in Figure 5A) that would be attained if the nascent chain assumed its native conformation throughout its synthesis. By the end of synthesis, the mean value of Q is only 0.13. This lack of appreciable structure suggests that confinement of the protein within the ribosomal tunnel—at least with the current simulation model—does not significantly promote native structure formation during synthesis. (Tests of the sensitivity of this result to the parameters of the simulation model are considered in a subsequent section.) Since the question of confinement effects on protein stability is of current interest [19,21,22], this issue was investigated further by performing an additional 50 coupled synthesis–folding simulations in which the ribosome structure was omitted, but which were otherwise performed in exactly the same way. If confinement within the tunnel promotes structure formation, the folding coordinate Q for the nascent protein during synthesis would be expected to be somewhat higher in the simulations performed in the presence of the ribosome than in those without the ribosome. The mean values of Q in the two sets of simulations are compared in Figure 5A, from which it is apparent that no such effect is observed. In fact, toward the end of synthesis, significantly higher Q values are observed in the simulations that omit the ribosome (open circles) since the protein is able to begin folding by doubling back onto its tethered C-terminus: for obvious steric reasons, this cannot occur when the protein is synthesized within the ribosome. Again, the same behavior is observed with barnase: confinement within the tunnel does not promote tertiary structure formation (Figure 5B).

A further interesting behavior can be discerned from a close examination of Figure 5A and 5B. Although the maximum attainable Q and the mean Q in simulations performed in the absence of the ribosome both increase monotonically as the nascent chain grows (with the former by definition reaching Q = 1 when the chain is completely synthesized), there are periods in the simulations performed in the presence of the ribosome in which the mean value of Q actually *decreases* slightly as the nascent chain grows. A more detailed view of the destabilization of the protein structure associated with these periods can be obtained by plotting the Q_res_ values of all individual residues as a function of the nascent chain length (see Figure 5C and 5D for CI2 and barnase, respectively). With both proteins, there are periods (marked by arrows) in which the Q_res_ values of several residues drop simultaneously as the chain increases in length; in most cases, the Q_res_ values recover again as the chain extends further in length. It is important to note that this decrease is not simply due to natural fluctuations in the amount of structure formation since the plotted values represent mean values averaged over 100-ns periods and over 30 independent trajectories. Supporting this contention is the fact that similar decreases do not occur at all in simulations in which the ribosome structure is omitted: in such cases, once increased, the Q_res_ values of all residues never undergo any significant decrease (Figure S3). This difference in behavior indicates, therefore, that it is the relative position of the nascent chain within the ribosome tunnel that is responsible for modulating the extent of its structure formation during synthesis.

### Kinetics of De Novo Folding and Refolding

**Figure 6:**
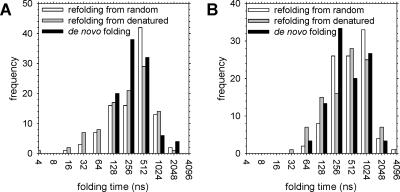
Simulated Kinetics of Refolding and De Novo Folding for CI2 and Barnase (A) Histograms of folding times obtained from refolding and de novo folding trajectories for CI2. (B) Same as (A), but for barnase.

For both proteins, the de novo folding behavior observed in the coupled synthesis–folding simulations was compared with that obtained in “refolding” simulations in the absence of the ribosome; refolding simulations were performed in two different ways: (1) starting from randomly generated conformations and (2) starting from denatured conformations (Q ~ 0.2) sampled from simulations performed at the folding temperature, T_fold_, of the proteins (i.e., where ΔG°_fold_ ~ 0). Histograms of the folding times obtained from the three types of simulations are shown for CI2 and barnase in Figure 6A and 6B, respectively. (Note that for de novo folding, we have chosen to measure time starting from the moment at which the final amino acid is released.) The distributions of folding times are in all cases reasonably well defined, suggesting that a meaningful sample of events has been obtained. There are three main results to note. First, as has already been alluded to, the simulated folding times (~300 ns) are orders of magnitude shorter than the real experimental folding times (~30 ms); this artificial rapidity of folding is an inevitable consequence of the extreme simplicity of the model (see Discussion). Despite the enormous disparity in absolute folding times, however, the relative refolding times of the two proteins obtained from the simulations are qualitatively correct: for CI2 and barnase, the median simulated refolding times are 280 ns and 395 ns, respectively, whereas the experimental folding times are 20 ms [39] and 50 ms [42], respectively. This result echoes a finding demonstrated more comprehensively by others, that simple Gō-type simulation models can correctly reproduce the relative folding rates of small proteins (e.g., [31]). Second, for both proteins, the median time for de novo folding is similar to the time taken for refolding from conformations sampled from the denatured ensemble, but is significantly shorter than for refolding from randomly generated conformations: for CI2, the median folding times for de novo folding, refolding from denatured, and refolding from random conformations, are 227 ns, 224 ns, and 280 ns, respectively; for barnase, the median folding times for de novo folding, refolding from denatured, and refolding from random conformations are 242 ns, 303 ns, and 395 ns, respectively. Interestingly, a Westenberg-Mood median test (http://www.fon.hum.uva.nl/Service/Statistics/Median_Test.html) indicates that the median folding times for de novo folding and for refolding from denatured conformations are statistically indistinguishable (*p*-values of 0.86 and 0.84 for the H_0_ hypothesis—that they are identical—for CI2 and barnase, respectively), but indicates that the median folding times for de novo folding and for refolding from random conformations *are* distinguishable (*p*-values of 0.23 and 0.15 for CI2 and barnase, respectively); the median folding times for refolding from random and denatured conformations are also distinguishable from one another (*p*-values of 0.12 and 0.12 for CI2 and barnase, respectively). These differences in median refolding times indicate (1) that the denatured conformations sampled at the folding temperature are decidedly non-random and (2) that the time required for a randomly constructed conformation to lose its “randomness” in refolding conditions is not negligible compared to the total time required to complete refolding in these simulations. A third and final result to point out is that for CI2, but not for barnase, there is a number of very fast refolding trajectories (folding times <100 ns) that are not observed in the case of de novo folding (Figure 6A); these fast trajectories are obtained regardless of whether the initial conformations for the refolding are randomly constructed or sampled from a denatured state ensemble at T_fold_. The absence of de novo folding times shorter than 100 ns appears to indicate that for CI2, there is a lower time limit for folding imposed by the time required to diffuse out of the ribosome tunnel; support for this interpretation comes from the fact that with the slower folder barnase, there are no refolding trajectories that are significantly faster than de novo trajectories (Figure 6B).

### Mechanisms of De Novo Folding and Refolding

**Figure 7:**
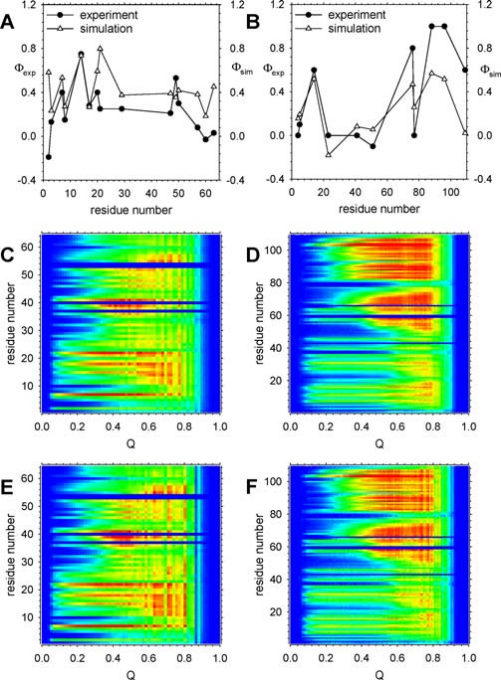
Simulated Mechanisms of Refolding and De Novo Folding for CI2 and Barnase (A) Comparison of computed and experimental Φ values for CI2. (B) Same as (A), but for for barnase. (C) Plot of Q_res_ versus Q for refolding of CI2. (D) Same as (C), but for refolding of barnase. (E) Same as (C), but for the post-synthesis stage of de novo folding of CI2. (F) Same as (C), but for the post-synthesis stage of de novo folding of barnase. In panels (C–F), Q_res_ values are shown on a continuous color scale between 0.0 (blue) and 0.75 (red) for CI2, and between 0.0 (blue) and 0.9 (red) for barnase.

The refolding mechanisms of CI2 and barnase have been the subject not only of very extensive experimental studies [42,43], but also of a large number of computational studies [44–48], including some that have employed Gō-type simulation models similar to that used here [29,30,49]. Since it is known that the simulated folding behavior obtained with Gō-type models can be surprisingly sensitive to the precise details of the model [49], it is worth verifying that the refolding mechanism obtained from the particular simulation model used here is reasonable by comparing it with experimental data. Probably the most useful experimental means of studying refolding mechanisms is “Φ-value analysis” [50,51] in which a residue's role in the transition state for folding is determined by comparing the effect of mutating the residue on the activation free energy for folding with its effect on the overall free energy of folding. Comparisons of computed Φ values with the corresponding experimental values are shown in Figure 7A and 7B for CI2 and barnase, respectively. It is clear from the comparisons that the computed Φ values are not in perfect quantitative agreement with the corresponding experimental values. Nevertheless, the overall agreement is significant, especially so given that the simulation model was not in any way parameterized to capture these data: the *R^2^* values of the correlations for CI2 and barnase are 0.37 and 0.77, respectively (the clearly rather poor correlation for CI2 is at least consistent with very recent results obtained by the Wolynes group using a similar simulation model [52]). Although the correspondence with experiment is not overwhelming, the simulation model does at least correctly capture the fact that for CI2, residues with intermediate Φ values (Φ ~ 0.4) are distributed throughout the primary sequence, while for barnase, residues with high Φ values—which are those that are most structured in the transition state ensemble—are located predominantly in the C-terminal β-sheet.

It is not straightforward to conduct a Φ-value analysis of the coupled–synthesis folding simulations (see Materials and Methods), nor would it be straightforward to do so experimentally. In order to provide an alternative means of comparing the mechanisms of de novo folding and refolding, therefore, the residue-specific folding coordinate Q_res_ discussed above was examined (results compiled in the remaining panels of Figure 7). Figure 7C shows how the mean Q_res_ values for all residues in CI2 change as a function of the *overall* Q value during refolding simulations (essentially identical results are obtained from examining refolding from both random and denatured conformations); note that data for Q greater than 0.8 are poor because each refolding trajectory is terminated as soon as folding to Q = 0.95 first occurs (Materials and Methods). Although these refolding simulations are performed under conditions in which the folded state of the protein is strongly thermodynamically favored, the view of the folding mechanism that emerges from this type of analysis is very similar to that obtained from the Φ-value analysis: in particular, at Q ~ 0.4, where the maximum in the plot of G versus Q occurs for the current model (Figure 1A), the more structured residues (red) correspond well with those showing higher Φ values in Figure 7A. More important, however, is the fact that this same type of analysis can also be applied to de novo folding trajectories, thus allowing us to investigate whether the simulated mechanisms for refolding and de novo folding differ significantly. Figure 7E shows how the mean Q_res_ values for residues in CI2 change as a function of the total Q value during the post-synthesis stage of the de novo folding simulation. It is immediately apparent from comparison of the two sets of data in Figure 7C and 7E that the simulated folding mechanisms for de novo folding and refolding of CI2 are essentially identical. (In fact, their correlation coefficient is 0.98.) The same qualitative results are obtained with barnase: (1) the view of the refolding mechanism that is obtained from a Q_res_ versus Q analysis is very similar to that obtained from Φ-value analysis, and (2) the simulated mechanisms of refolding and de novo folding are more or less identical (Figure 7D and 7F). For both of the small model proteins studied with the present simulation model then, the gradual, vectorial synthesis of the protein within the ribosome does not appear to effect any significant change in the folding mechanism.

### Coupled Synthesis and Folding of Artificially Stabilized CI2

**Figure 8:**
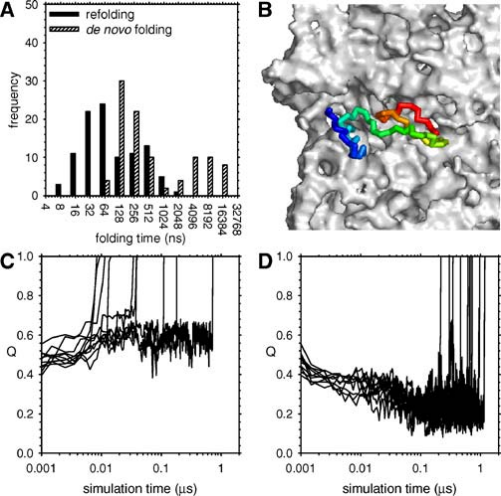
Consequences of Excessively Stabilizing CI2 (A) Histograms of folding times obtained from refolding and de novo folding trajectories of CI2 with ɛ = 0.80 kcal/mol. (B) Snapshot of a “trapped” trajectory. (C) Plots of Q versus time for ten previously trapped trajectories when restarted without the ribosome. (D) Same as (C), but restarted with the ribosome and ɛ = 0.60 kcal/mol.

The above discussion has focused on the folding behavior of proteins whose energy parameters have been calibrated to reproduce the experimental free energy of folding of the full-length proteins; in these simulations it is clear that both CI2 and barnase fold essentially post-translationally. Since it was of interest to consider what might happen if a protein began to fold significantly *during* synthesis, additional sets of simulations were performed in which the energy parameter for CI2 was deliberately exaggerated (ɛ = 0.80 kcal/mol) to increase the folded state's stability: with this parameter, the smaller CI2 fragments discussed above are all largely folded at 300 K (unpublished data). In simulations performed with this energy parameter, refolding of the full-length protein becomes, as expected, notably faster: the median time for refolding from random conformations decreases from approximately 280 ns to approximately 50 ns (Figure 8A). In contrast, in the coupled synthesis–folding simulations, the distribution of de novo folding times becomes strikingly bimodal, with median folding times of approximately 130 ns and approximately 4.5 μs for the two sub-populations (Figure 8A). The faster folding sub-population comprises 67% of the trajectories and, as was observed with the more realistic energy parameter for CI2, appears to be limited by the time taken for the protein to diffuse out of the ribosome tunnel. The slower folding sub-population is more interesting: it comprises folding trajectories in which the protein forms significant tertiary structure while still positioned within the tunnel. With the present simulation model, the tunnel is not sufficiently wide to allow complete folding to occur, but it does allow significant partial structure formation (Q ~ 0.5), and since there is a constriction at the tunnel's exit [53], the partially folded protein is prevented from diffusing out into solution to continue folding (e.g., see Figure 8B; see Figure S4 for snapshots from a typical trajectory). These partially folded molecules become stuck for significant periods of time, and in fact three of the 50 trajectories failed to escape and fold completely even 10 μs after the completion of synthesis, which is 200 times greater than the median time for refolding simulated in solution.

Additional “control” simulations were performed on ten of these “stuck” simulations, starting from partially folded conformations that were formed at a point 7 μs into the simulation (i.e., 1 μs after synthesis was completed). First, the ten simulations were restarted with the same exaggerated energy parameter but *without* the ribosome (Figure 8C). All ten molecules rapidly complete folding, which indicates, as might be expected, that the partially folded structures formed within the tunnel are not significantly “off pathway” with regard to formation of the final native structure. More interestingly, in these restarted simulations, Q does not initially decrease following removal of the ribosome; this provides further evidence that the elements of native tertiary structure are not appreciably stabilized by confinement within the tunnel.

As a second control, the ten “stuck” simulations were restarted with the ribosome retained but using the energy parameter (ɛ = 0.60 kcal/mol) that realistically describes the experimental ΔG°_fold_ for CI2. Interestingly, the pre-existing tertiary structure in these simulations rapidly melts (Figure 8D), and the proteins diffuse out of the ribosome and complete their folding in the solution environment. (Snapshots of a typical trajectory are provided in Figure S5.) This is an important result since without it, one straightforward explanation that could be proposed for the fact that significant structure does *not* form in simulations that use the realistic energy parameter from the very beginning (Figure 5A) would be that synthesis is simply conducted too fast in the simulations for partially folded conformations to be adopted. Although one counterargument to this would be to note that care has been taken to realistically match the rates of synthesis and folding in the simulations (Materials and Methods), Figure 8D provides a perhaps more compelling counterargument: it suggests that even if partially folded conformations were to form within the tunnel with the realistic energy model (ɛ = 0.60 kcal/mol), they would be unlikely to be sufficiently stable to persist or to cause a decrease in the rate of de novo folding. A still more explicit test of the appropriateness of the timescale of synthesis applied in the simulations is described in a subsequent section.

### Coupled Synthesis and Folding of SFVP

**Figure 9:**
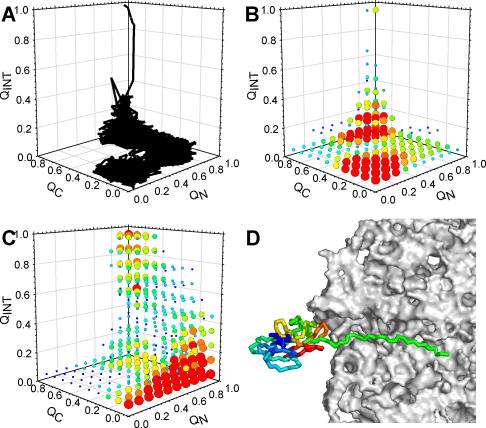
Folding of the Two-Domain Protein SFVP (A) Evolution of Q_N_, Q_C_, and Q_INT_ during a typical refolding trajectory of the serine protease module of SFVP. (B) The most populated points in (Q_N_, Q_C_, Q_INT_) space during 100 refolding trajectories. Each point in the (Q_N_, Q_C_, Q_INT_) space is counted only once per trajectory in order to prevent repeated revisiting of the same region in a trajectory from biasing the results. Symbol size and color reflect relative population (large, red symbols being populated in 100% of simulations). (C) Same as (B), but for ten coupled synthesis–folding simulations. (D) Snapshot of the moment that native structure is cotranslationally formed; the green chain extending back to the peptidyltransferase active site represents the approximately 28 residues synthesized after the protease module's final residue.

A final series of simulations was performed to investigate the coupled synthesis–folding of the two-domain serine protease module of the Semliki forest virus protein (SFVP): this 149-residue module has been shown to autoproteolytically cleave itself from the rest of the 1,257-residue protein during translation, which, since the protease acts only in *cis,* indicates that the module must fold to its native state cotranslationally [54]. Since it proved computationally intractable to calibrate the energy parameter (ɛ) for SFVP so that the experimental ΔG°_fold_ was reproduced, three sets of simulations were performed, each with a different energy parameter (Figure S6). In Figure 9, the results obtained with ɛ = 0.54 kcal/mol are shown since they produced a median refolding time (~677 ns) that was approximately consistent with the relative experimental refolding times of SFVP and CI2 (50 ms [55] and 20 ms [39], respectively); however, similar behavior was observed with all three parameter values studied (see below). A convenient way of following folding in a two-domain protein is to independently monitor folding coordinates for the N-terminal domain (Q_N_), the C-terminal domain (Q_C_), and the interface region (Q_INT_) (Materials and Methods). Figure 9A shows how the values of Q_N_, Q_C_, and Q_INT_ evolve during a typical refolding trajectory of SFVP (snapshots are shown in Figure S7). Starting with Q_N_ ~ Q_C_ ~ Q_INT_ ~ 0, the N-terminal domain (in this particular trajectory) folds first (Q_N_ ~ 0.8) with both Q_C_ and Q_INT_ remaining close to their initial values. Following this, the C-terminal domain folds independently, and finally, the two domains associate and the interfacial contacts form. Figure 9B shows the density of points in the three-dimensional Q_N_, Q_C_, Q_INT_ space sampled during 100 refolding simulations, with the more densely populated points colored red. In the simulations, refolding occurs one domain at a time (with the N-terminal domain being marginally more likely to fold first) with association of the two folded domains being the final step.

**Figure 10:**
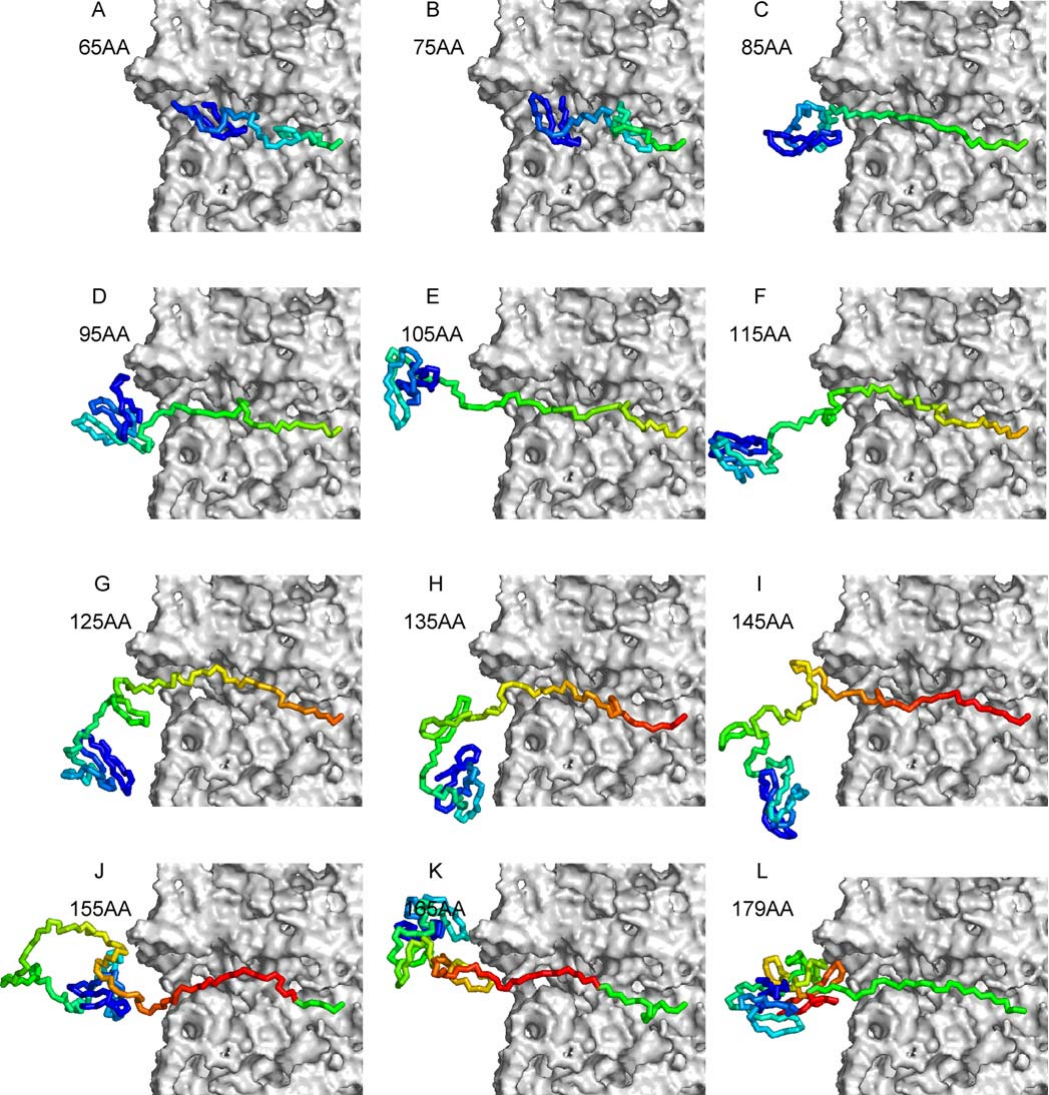
A Typical Coupled Synthesis–Folding Simulation of SFVP Snapshots from a typical de novo folding simulation of SFVP in the presence of the ribosome (grey surface). The nascent chain is colored from blue (N-terminus) to red (C-terminus), with its length (in amino acids) indicated in each panel. This figure was prepared with PyMOL[95].

Intriguingly, the folding behavior observed in the coupled synthesis–folding simulations is quite different (Figure 9C). In the simulations, the N-terminal domain shows a much more pronounced tendency to fold first, and both the folding of the C-terminal domain *and* the formation of the domain–domain interface show a tendency to occur simultaneously and in concert with synthesis. (See Figure 10 for snapshots of a typical trajectory and Figure S8 for all trajectories in Q_N_, Q_C_, Q_INT_ space.) This difference between refolding and de novo folding becomes more pronounced with ɛ = 0.57 kcal/mol (Figures S9 and S10). A structural snapshot of the protein at the moment at which it attains its native structure, with the final crucial residue trp149 (trp267 in the numbering system of the full-length protein) folded into the enzyme's active site, is shown in Figure 9D. In ten coupled synthesis–folding simulations, the mean length of the nascent protein when the native structure of the serine protease module is attained is 180 ± 5 residues, indicating that an additional 31 residues must be synthesized before the nascent protein is capable of autoproteolysis. This number is somewhat shorter than the 43 residues estimated on the basis of in vivo experiments [54], and this is perhaps attributable to the limited structural resolution of the current model and the fact that simulations are terminated as soon as the final residue is in place, an action that in effect corresponds to assuming that proteolysis occurs instantaneously.

### Sensitivity of Results to Parameters

**Figure 11:**
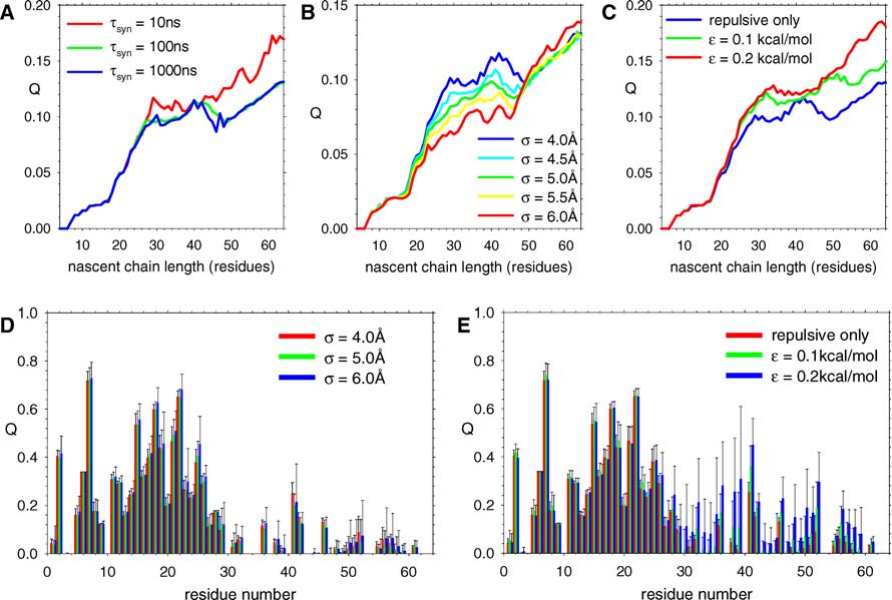
Parameter Sensitivity of Coupled Synthesis–Folding Simulations of CI2 (A) Mean values of Q during coupled synthesis–folding of CI2 comparing the effects of using different timescales for amino acid synthesis (τ_syn_). (B) Same as (A), but comparing the effects of assigning larger radii to ribosome pseudo-atoms. (C) Same as (A), but comparing the effects of including energetically favorable interactions between the nascent protein and the ribosome. (D) Distribution of mean Q_res_ values obtained during the synthesis of the final amino acid, comparing the effects of assigning larger radii to ribosome pseudo-atoms; error bars indicate the standard deviation of values obtained from 30 independent trajectories. (E) Same as (D), but comparing the effects of including energetically favorable interactions between the nascent protein and the ribosome.

Since a number of the parameters of the current simulation model could reasonably be assigned a range of values, additional simulations were performed to investigate the robustness of some of the behavior(s) described above; the results of these studies are compiled in Figure 11. As noted above, one important parameter to investigate is the rate at which new amino acids are “synthesized” in the coupled synthesis–folding simulations. As discussed in Materials and Methods, an attempt to match the simulations' relative rates of synthesis and folding with those of experiments suggests that a reasonable rate of addition is one amino acid per 100 ns, and this is, therefore, the rate that has been used throughout all simulations discussed up to this point. To test the effects of both faster and slower rates of synthesis, however, ribosome-mediated simulations of CI2 were performed with amino acids added at a rate of one per 10 ns and at a rate of one per 1,000 ns. Plots of the mean folding coordinate Q as a function of time for these three sets of simulations are shown for the period during synthesis in Figure 11A. From this it can be seen that the plots obtained for synthesis times of 100 ns and 1,000 ns are essentially identical, suggesting that the rate of synthesis in the former case is already sufficiently slow that it allows complete or near-complete configurational sampling and that use of a much slower synthesis rate (which would dramatically decrease the number of simulations that could be performed with a given amount of computer time) is unnecessary. Interestingly, however, the plot obtained for a synthesis time of 10 ns differs significantly—and in such a way that the formation of tertiary structure during synthesis is actually increased. This latter effect appears to result from amino acids being synthesized more rapidly than they can diffuse out of the ribosome tunnel, with the resulting transient increase in the local concentration of residues promoting the formation of residue–residue contacts.

A second aspect that was investigated was the effect of increasing the radius of the ribosome pseudo-atoms: doing so effectively further constricts the dimensions of the ribosome tunnel and therefore allows potential confinement effects to be magnified. Figure 11B plots the mean folding coordinate Q as a function of time for sets of simulations in which σ_ij_ for nascent protein–ribosome interactions (see Materials and Methods) ranged between 4.0 and 6.0 Å. (Complete comparisons showing error bars are given in Figure S11.) It is apparent from these plots that increasing the degree of confinement within the tunnel decreases the extent of tertiary structure formation during the early stages of synthesis (when the nascent chain is 20–40 residues long), but by the final stages of synthesis, it appears to make little difference. A comparison of the individual Q_res_ values computed for each residue during the synthesis of the final amino acid indicates that the overall structure of the protein at this point of the simulation is unaffected by the increased size of the ribosome atoms (Figure 11D). As might therefore be expected, a comparison of Q_res_ versus Q plots for the period following synthesis indicates that the overall de novo folding mechanism is essentially unchanged (Figure S12). There is, however, a suggestion that for the most confined case (σ_ij_ = 6.0 Å), the formation of the native structure is somewhat slowed (the *p*-value for the H_0_ hypothesis that the de novo median folding times obtained with σ_ij_ = 4.0 and 6.0 Å are identical is less than 0.44): this almost certainly reflects the increased difficulty that the newly synthesized chain encounters in “wriggling free” of the ribosome.

A third aspect that was investigated was the effect of including favorable energetic interactions between the nascent protein chain and the ribosome (see Materials and Methods). In this case, the inclusion of progressively favorable interactions increases the level of tertiary structure formation during *all* stages of synthesis (Figure 11C; complete comparisons showing error bars are given in Figure S11); this result presumably reflects the less-confined environment experienced by the nascent chain when its repulsive interactions with the walls of the tunnel are replaced by attractive interactions. Examination of the Q_res_ values of all residues shows that the increase in the overall Q during the synthesis of the final residue (Figure 11E) is due primarily to increased structure in the C-terminal residues (residues ~30 and up). There is also a significant slowing down in the rate of de novo folding when attractive interactions are included (the median folding time increases from 227 ns without attractive interactions to approximately 570 ns in the most attractive case), suggesting, perhaps not surprisingly, that some sticking of the nascent chain to (or within) the tunnel does occur. As was observed in the case of increasing the ribosome atom radii, however, no dramatic change of the de novo folding mechanism occurs in the post-synthesis stage of the simulations (Figure S12).

A final aspect that was investigated was the effect of altering the balance between local and non-local interactions in the protein energetic model. As noted in Materials and Methods, the adjustment of the energy parameter ɛ to match the experimental folding free energy was made in such a way as to keep constant the ratio of the (local) dihedral angle barrier heights to the (non-local) native non-bonded interaction well depths. It is, of course, possible to match the experimental folding free energy with different weights applied to the local and non-local terms: a weakened set of non-bonded Gō interactions can be compensated for by strengthening the dihedral angle terms, which drive the chain to adopt the correct local secondary structure. All CI2 simulations discussed up to this point assigned a barrier height of 0.50 kcal/mol to the dihedral potential V_1_ (Materials and Methods), but to investigate the sensitivity of results to this parameter, additional sets of simulations were performed with V_1_ = 0.125, 0.25, and 1.0 kcal/mol respectively. The results of these simulations are compiled in Figure S13 from which it can be seen that the use of alternative parameter sets does not significantly affect any conclusions drawn here concerning ribosome-mediated folding: in particular, in all cases, confinement within the ribosome results in a general suppression of structure formation relative to what is observed in absence of the ribosome, but there are periods during synthesis where Q undergoes a clear though transient decrease. It is encouraging to see that the parameter set used in most of the simulations reported here (V_1_ = 0.5 kcal/mol) produces the most cooperative folding behavior of all parameter sets tested (Figure S13A and S13B).

## Discussion

As always with work based entirely on the use of computer simulations, it is not only important to test the effects of changing parameter values (see previous section), it is also necessary to state and evaluate the assumptions and approximations that are implicit in the approach. A first key assumption of the present work is that a rather simple simulation model developed to study protein refolding events can be extended and applied to model the more complex phenomenon of coupled synthesis and folding within the large ribosomal subunit. The limitations of Gō-type models are well known (for an extended discussion see Text S1), and the impact of these limitations is clearly felt in some of the aspects considered here: the close examinations of structure in the fragments and the only rough agreement between the computed and experimental Φ values both indicate that specific details of the folding mechanisms and equilibria are not perfectly reproduced by the model. These drawbacks are, however, to be considered in the context of the method's significant advantages, not least of which is the fact that it is its computational simplicity that makes the rather wide range of simulations discussed here feasible with usual computational resources. Moreover, the new *refolding* results presented here—indicating that increases in stability and folding cooperativity with increasing fragment lengths are correctly captured—provide further demonstrations that this very simple computational model can give surprisingly robust descriptions of real protein behavior (but see Text S1 for more on this subject). The quite close agreement between the mean computed Q values of fragments and the fluorescence intensities measured experimentally by the Fersht group (Figure 1C and 1D) seems especially notable. It is, of course, important to recognize that such a comparison assumes that the fluorescence intensity provides a meaningful measure of how “folded” each fragment is, whereas in reality it simply reports on the local environment experienced by the fluorophores (as well as their number), but for both model proteins studied here, this appears to be a reasonable assumption [34,36]. In the same vein, it is perhaps worth noting that the somewhat less-successful description of *local* secondary and tertiary structure formation for these proteins might also stem in part from a similar disconnect between the simulation and experimental observables: CD spectra for example are a composite of contributions from multiple structural elements, and do not, therefore, always lend themselves to direct comparisons with computed secondary structures [34,36]; this is especially likely to be an issue with barnase since the native protein's far-ultraviolet circular dichroism spectrum does not correspond with expectations based on its known secondary structure [56]. As a final note on the subject of fragment stabilities, it is worth mentioning that the Cavagnero group has proposed using differences in solvent-accessible surface areas as a means of predicting the relative stabilities of N-terminal fragments [57].

A second issue to be considered is the extent to which the results discussed here might depend on the choice of the reaction coordinate used to monitor folding. The subject of whether the fraction of native contacts, Q, represents a good reaction coordinate continues to attract a considerable amount of discussion in the literature, and a question of particular interest is whether finding the maximum in the plot of free energy versus Q provides a safe way of identifying those protein conformations that are members of the transition state ensemble (TSE) for folding. Although making this assumption is very convenient—and was used here to calculate the Φ values shown in Figure 7A and 7B—it has been argued by Shakhnovich, Pande, and others that the members of the TSE should instead be identified by explicitly demonstrating that they have a probability of proceeding to the folded state (P_fold_) of 0.5 [46,58,59]. Wolynes and coworkers on the other hand have argued that the P_fold_ approach can itself lead to difficulties when applied to more-complex folding mechanisms [52], and have shown that for a few test cases (including CI2), there is in any case a reasonable correspondence between the two approaches. This issue has also been considered in the present work for both CI2 and barnase: explicit computation of P_fold_ for a large number of conformations segregated by their Q values indicates that those with Q values corresponding to the maximum in the plot of G versus Q do have on average a P_fold_ of approximately 0.5 (Figure S14; it should also be noted, however, that the distribution of P_fold_ values does not center on this value, but instead is bimodal). While this correspondence provides some support for the present work's use of Q as the primary descriptor of the extent of folding, two additional points should also be remembered. First, throughout this work we have taken care to compare the folding behavior observed in coupled synthesis–folding simulations performed in the presence of the ribosome with either (1) refolding from random or denatured conformations, or (2) coupled synthesis–folding simulations performed in the absence of the ribosome: in all of these comparisons, identical reaction coordinate definitions have been used, so at least some of the concerns that might remain about an inappropriate choice of reaction coordinate would be expected to cancel out. Second, any over-reliance on Q has been mitigated where practical by extending discussion of the simulated folding mechanism to consideration of Q values for individual residues (Q_res_), thus allowing a considerably more detailed view of the mechanism to be obtained.

A third issue to discuss concerns the simulated timescales of both protein folding and protein synthesis. The artificially rapid folding that occurs in the simulations is a consequence of the huge simplification of the energy landscape that attends the structural assumptions made (i.e., the C_α_-only protein model) and its barrier-free description of residue–residue interactions. A closer connection between the simulated and experimental timescales of folding is to be expected in (atomically detailed) simulations of protein folding events that do not make such assumptions, such as the massive distributed computing simulations pioneered by the Pande group [60,61] and those that specifically address very fast folding reactions (e.g., [62]). Recognition of the disconnect between the simulated and experimental timescales appears to be one reason why many simulation studies that use structurally simplified models report folding kinetics only in terms of reduced units (such as simulation “steps”); in our view, however, this tends to obscure what is actually a real difference, so the nanosecond–microsecond timescale of the simulations has been identified explicitly here. Of course, it should be remembered that for most purposes, the discrepancy between simulated and experimental timescales is unimportant since *relative* folding rates can still be directly compared with one another—often with extremely good results (e.g., [31]).

In the present context, however, the disparity is potentially of greater significance since there is a second key timescale that must also be considered if the simulation model is to behave realistically: this is the rate of protein synthesis. It is, therefore, important to recall that all coupled synthesis–folding simulations reported here were conducted with the rate of synthesis explicitly matched with that of folding so that their relative rates are consistent with experiment (see Materials and Methods). That said, it should also be noted that this matching has been done assuming that amino acid synthesis occurs at a constant rate; in reality, however, there can be significant variations (up to 25-fold) in translation rates, depending on the availability of the appropriate cognate tRNAs (e.g., [63]), the nature of the base-pairing interaction between codon and anticodon (e.g., [64,65]), and/or the presence of secondary structure in the coding mRNA (e.g., [66]). There has been some discussion in the literature that slowly translated regions may, in general, occur at boundaries between protein domains (e.g., [67,68]), and this issue could be investigated in future for proteins in which the effects of different codons on translational pausing have been studied (e.g., [69]). For the more limited goals of the present work, however, the comparison shown in Figure 11A suggests that the choice of a (constant) simulated rate of one amino acid per 100 ns at least provides a reasonable compromise between the need to allow the nascent chain to sample a wide range of conformations during synthesis and the need to make the simulations sufficiently rapid that many independent trajectories, performed under many different simulation conditions, can be obtained.

A final issue that needs to be addressed is the level of structural detail employed in the model of the nascent protein and the nature of its interaction with the ribosome. It is clearly a major simplification to strip away the sidechains, to ignore non-native interactions within the protein, and to overlook (in most of the simulations reported here) the possibility of favorable energetic interactions between the nascent chain and the ribosome. Before dismissing the entire body of work, however, it should be remembered that all of these assumptions—unpalatable as some of them undoubtedly are—have been made in the interests of computational feasibility. The simulations in which increasing radii were assigned to the ribosome atoms (Figure 11B) can be thought of as crudely mimicking what might happen if the nascent protein was made more bulky by the inclusion of its sidechains, but they are, of course, unlikely to be a perfect substitute for simulations in which sidechains are explicitly represented. Developing a model of the latter type is, therefore, a clear and important goal for the near future. That said, it should not be assumed that a (properly parameterized) protein model in which sidechains are explicitly represented would automatically lead to a more realistic view of cotranslational folding: it may be, for example, that one would then also need to explicitly model the conformational dynamics of the ribosome itself in order for the now-bulkier protein to diffuse out of the tunnel. Although there have already been studies reported in the literature in which the conformational dynamics of the ribosome have been modeled (e.g., [70–72]), including such dynamics while modeling protein synthesis would likely dramatically increase the computational burden associated with the simulations.

With all of the above issues in mind, it is worth reiterating that the coupled synthesis–folding simulations have produced three main results. First, for the comparatively simple proteins CI2 and barnase, the simulated de novo folding mechanisms are more or less identical to those for refolding, and the folding kinetics are effectively indistinguishable from those for refolding from conformations sampled from the denatured ensemble. Although it might be argued that the same conclusion could be reached simply on the basis of the known lower stability of N-terminal fragments [33], the simulations reported here place this reasoning on a firmer footing by showing that confinement of the nascent protein within the ribosome tunnel during the early stages of its synthesis does not appear to significantly affect its folding. The present results, therefore, provide support for the use of in vitro refolding experiments as models of in vivo folding for simple globular proteins.

The fact that confinement of the nascent protein chain within the ribosome tunnel does not appear to promote structure formation deserves further comment, not only because of the current interest in confinement effects in general, but also because of its connection with important recent experimental work. The fluorescence-resonance energy transfer (FRET) experiments conducted by the Johnson group [73,74] and the pegylation-based “molecular tape measure” experiments performed by the Deutsch group [75,76] have both provided strong evidence that a significant structural compaction of nascent peptides can occur within the ribosome tunnel; although not directly demonstrated, the most straightforward interpretation of these experiments is that the nascent peptides assume helical conformations. Especially interesting are the Deutsch group's recent results [76] indicating that the extent of compaction can vary significantly depending on the location within the tunnel; a thermodynamic model was used to estimate that the contribution made by the ribosome to structure formation was up to 1 kcal/mol. The *cause* of this stabilization of compact structure is more difficult to establish, and possible factors suggested by the authors included interactions between the nascent peptide and non-polar sidechains in the tunnel, locally altered water activity modulating the hydrophobic effect, and confinement effects.

The latter idea has been investigated by Thirumalai and colleagues with a simple cylindrical model for the ribosome tunnel, and has led them to the conclusion that significant stabilization of an α-helix can occur depending on the diameter assigned to the cylinder [22]. In contrast, the present simulations suggest that for the particular sequences studied here (which adopt predominantly β-sheet conformations), confinement *alone* does not cause appreciable changes in the degree of structure formation. It should be noted that the present simulations use a much more detailed model of the ribosome tunnel than that used by the Thirumalai group, and focus on (quite different) proteins for which the simulated description of fragment stabilities has been shown to be in good agreement with experimental data (Figure 1C and 1D). It is also important to note that the present simulations correctly capture the Deutsch group's finding that the extent of structure formation can both increase and decrease depending on the length of the nascent chain (Figure 5C and 5D), although since the proteins studied here are different from those investigated experimentally, it is not possible to make a direct comparison between the two studies. It seems likely, in fact, that this type of behavior may turn out to be quite dependent both on the nature of the nascent protein's sequence and its native structure.

The second main result of the coupled synthesis–folding simulations is that in contrast to what is seen with the simpler model proteins CI2 and barnase, for the two-domain protease module of SFVP, the simulated de novo folding and refolding mechanisms differ significantly. In the simulations, refolding occurs primarily by independent folding of the N- and C-domains—with the former being slightly more likely to fold first—followed by association of the two folded domains. The de novo folding pathway, on the other hand, often occurs by gradual accretion of C-domain structure onto a pre-folded N-domain (Figures S8 and S9). The view of refolding obtained from the simulations is only partly consistent with the interpretation of recent experiments on SFVP's refolding kinetics [55], although given that the latter studies have not yet been extended to give the more structurally resolved view obtainable from Φ-value analysis [50,51], it is not yet certain that the experimental and simulation results are actually inconsistent with one another. The more important point here, however, is that the differences observed between the de novo folding and refolding mechanisms of SFVP suggest that, while the folding mechanisms for the two scenarios might be identical for single or isolated domains, the same may not be true for the assembly of multi-domain proteins.

The final and perhaps most interesting set of results obtained from the de novo folding simulations is that shown in Figure 8: with an artificially stabilized version of CI2, the rate of folding (or perhaps more correctly, the rate of adopting the native conformation) is severely compromised for a significant fraction of the molecules because of entrapment of partially folded conformations in the ribosome tunnel. It is important to note that this effect does *not* result from any attractive interaction between the atoms of the protein and the ribosome, since in the simulations discussed, all such interactions are purely repulsive. Instead, it is the protein—by stubbornly refusing to transiently unfold when faced with a constricted exit—that prevents its own escape. There has already been some discussion in the literature that cooperativity in protein folding might be evolutionarily advantageous [77], and the present results provide an intriguing additional argument to this effect: the premature (non-cooperative) acquisition of stable tertiary structure within the ribosome tunnel could hinder the rapid release and folding of individual protein domains. Of course, this is not to exclude the possibility that smaller elements of (secondary) structure might form: as noted above, there is now considerable evidence for conformational preferences being expressed in short nascent sequences [14,73–76,78]. In addition, electron density observed in recent cryo-electron microscopy studies also indicates a degree of structure formation in the tunnel [79].

In closing, it should be noted that there is clearly a number of complexities to the behavior of nascent protein sequences within the ribosome that are not captured in the present simulation model. Nascent sequences can regulate translation [80] through direct interference with the peptidyltransferase site or by interaction with proteins further down the tunnel, and FRET-based studies have indicated that these interactions can cause far-reaching conformational changes in the ribosome [81]. Extension of the present simulation model to describe some of these complexities should eventually prove possible: in addition to overcoming some of the basic structural and energetic assumptions discussed above, it will be important to develop a more realistic description of the process of synthesis that incorporates a mechanism for feeding back binding events within the tunnel to the peptidyltransferase site. Future studies along these lines should have the potential to provide considerable new insights into both the mechanism and regulation of protein folding in vivo.

## Materials and Methods

### The protein model.

The structural and energetic model used in the simulations reported here is essentially identical to the Gō model employed by Clementi, Onuchic, and others [29–32]. The protein of interest is modeled at the residue level, i.e., each residue is represented only by its C_α_ atom, and standard molecular mechanics terms are used to model bonded interactions (pseudo-bond stretches, pseudo-bond angle deformations, and pseudo-dihedral angle rotations) between contiguous residues. The contribution to the energy of any conformation of the protein from these molecular mechanics bonding terms is:

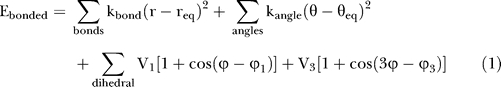
where r, θ, and ϕ refer to the bond distances, bond angles, and bond dihedrals, respectively, r_eq_ and θ_eq_ are the corresponding bond distances and angles in the native state structure, and ϕ_1_ and ϕ_3_ are phase angles defining the position of the energy maxima of the cosine terms. In all simulations reported here, the force constants k_bond_ and k_angle_ were set to 100 kcal/mol/Å and 20 kcal/mol/rad, respectively; the setting of the potential barriers for the dihedral terms (V_1_ and V_3_) is discussed below.

Interactions between non-bonded residues (those separated by three or more residues) are represented by a Gō model [28,82], in which one of two different energy functions is applied depending on whether the two residues form a direct contact in the native state structure. Residue pairs that form a contact in the native state are modeled using a Lennard-Jones–like potential:

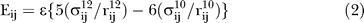
where ɛ is the energy well depth assigned to the contact (see below), and r_ij_ and σ_ij_ are the distances between the two C_α_s in the current and native state structures, respectively. Residue pairs that are not in contact in the native state are modeled with a purely repulsive term:

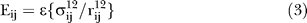
with σ_ij_ in this case being set to 4 Å. In the present work, two residues were determined to form a contact in the native state if any of their atoms were within 4.5 Å of each other after hydrogens had been added to the structures using WHAT IF [83]. The number of native contacts determined in this way for CI2 (using the Protein Data Bank (http://www.rcsb.org) pdb file 2CI2; [84]), barnase (pdb file 1RNB; [85]), and the serine protease module of the SFVP (pdb file 1VCP; [86]) were 169, 326, and 509 respectively.

The energetic parameters ɛ, V_1_, and V_3_ were initially set to 0.6, 0.5, and 0.25 kcal/mol, respectively, because these parameters approximately reproduced the experimental folding free energy of CI2 in water at 25 °C, ΔG°_fold_ = −7 kcal/mol [39]. For barnase, a series of simulations was performed in which the three parameters were all scaled by a single factor until the experimental ΔG°_fold_ of −9 kcal/mol [40] was approximately reproduced: the optimized values selected in this way were 0.57, 0.475, and 0.2375 kcal/mol, respectively. Scaling the three energy terms by a single factor is an attempt to maintain an energetic balance between the non-local interactions (controlled by ɛ) and the local interactions (controlled by V_1_ and V_3_): previous work has shown that this balance can significantly affect simulated folding behavior [87]. For the considerably larger serine protease module of SFVP, computational expense prevented systematic optimization of the energy parameters to reproduce ΔG°_fold_. Instead, simulations were performed with a range of values ɛ = 0.54, 0.57, and 0.60 kcal/mol (with V_1_ and V_3_ again being scaled by the same factor to maintain the balance of non-local and local terms). As described in Results, a series of simulations of CI2 was also performed with parameters that deliberately exaggerated its stability: the values of ɛ, V_1_, and V_3_ in these simulations were 0.8, 0.6667, and 0.3333 kcal/mol, respectively. Finally, in order to test the effects of altering the balance between local and non-local interactions in the protein model, 30 independent simulations of CI2 were also performed with each of three additional alternative sets of parameters tuned to match the experimental ΔG°_fold_ of CI2 at 300 K (see Results, Figure S13); the values of ɛ, V_1_, and V_3_ in these three sets were (1) 0.7795, 0.125, and 0.0625 kcal/mol, (2) 0.7121, 0.25, and 0.125 kcal/mol, and (3) 0.4604, 1.0, and 0.5 kcal/mol.

### The simulation algorithm.

The time-dependent conformational behavior of each protein of interest was simulated with the Brownian dynamics (BD) algorithm developed by Ermak and McCammon [88] and implemented in software written by the author. Each C_α_ pseudo-atom was assigned a diffusion coefficient of 0.1 Å^2^ ps^−1^, and an integration timestep of 25 fs was used throughout. To accelerate the simulations, interactions between nonbonded residues were only computed when they were within 10 Å (for non-native residue pairs) or σ_ij_ + 6 Å (for native residue pairs); the residue pairs satisfying these distance requirements were entered in a list that was recomputed every 20 integration steps. After each integration step, the pseudo-bonds between contiguous C_α_ atoms were constrained to their native lengths using the LINCS algorithm [89].

### Computing the thermodynamics of folding.

Following others (e.g., [29,30]), the extent of folding of a protein at any point in a simulation was quantified simply in terms of the fraction, Q, of native residue pairs that were currently in contact (i.e., within a distance of 1.2 σ_ij_). Using this definition, Q = 0 corresponds to a protein conformation so completely unfolded that it contains no native contacts at all, whereas Q = 1 indicates a fully folded protein; Q is therefore often referred to as an “order parameter.” For both CI2 and barnase, calibration of the energy parameters (see above) was achieved by performing simulations of 100-μs length near the folding temperature T_fold_, (i.e., the temperature at which ΔG°_fold_ = 0), and computing the free energy at 300 K using now-standard histogram techniques [90,91]. The same histogram techniques were used to compute the folding free energy surface at all temperatures from 200 K to 400 K. Folding free energy surfaces for *fragments* of CI2 and barnase were computed from additional 100-μs simulations in the same way, using the optimized energy parameters obtained for the full-length proteins.

### Computing Φ values and assessing Q as a reaction coordinate.

Experimentally, Φ values are obtained by comparing the effects of a single-residue mutation on the kinetics and thermodynamics of the wild-type protein's folding. Computationally, simulating the refolding kinetics of a large number of mutants directly would be a very expensive undertaking since it would require many individual trajectories to be computed for each mutant. To avoid this computational expense, the kinetic component of the Φ value can instead be calculated from the same one-dimensional folding free energy landscape used to obtain the thermodynamic component of the Φ value, by examining the effects of a mutation on the height of the free energy barrier to folding at T_fold_. This approach requires only that the folding free energy landscape for each mutant be computed, which as discussed above, can be achieved by performing 100-μs simulations. In the present study, mutations to alanine were modeled simply by removing all atoms of a sidechain beyond the C_β_ atom and re-computing the set of Gō-interacting residue pairs in the same way as done for the wild-type protein. Since in a number of cases this removal of the additional atoms did not result in any change of the list of Gō pairs, it was not possible to compute Φ values for these mutants (since their energies would be identical to those of the wild-type protein). Where possible however, Φ values were computed with the usual expression [50,51] Φ = ΔΔG°^U−‡^/ΔΔG°^U−F^ where ΔΔG°^U−‡^ represents the change in free energy difference between the unfolded state (U) and the “transition state” (‡) for folding, and ΔΔG°^U−F^ represents the change in the free energy difference between the unfolded (U) and folded (F) states.

An implicit assumption in the above approach is that the maximum in the plot of G versus Q adequately represents the transition state such that structures with this value of Q have the same probability of proceeding to the folded state as they do of proceeding to the unfolded state ensemble. This assumption was tested explicitly in the following way. Structural snapshots were first sorted into bins based on their Q value (with bin widths of 0.025 units). From those bins of interest, 500 snapshots were randomly selected, and each selected snapshot was used as a starting point for computing 20 independent folding trajectories. Trajectories were continued until the either the folded or unfolded state ensembles were reached: for CI2, trajectories were terminated when Q < 0.175 or Q > 0.875 was reached; for barnase, trajectories were terminated when Q < 0.275 or Q > 0.825 was reached. For each bin of Q values, therefore, a total of 10,000 folding trajectories were simulated, from which the probability of folding (P_fold_) as a function of Q could be reliably computed.

### Measurements of secondary structure in fragments.

In order to measure the extent of secondary structure formation in all fragments of CI2 and barnase studied, 10,000 structural snapshots (taken at intervals of 10 ns) were analyzed for each fragment at its folding temperature T_fold_. Since our structural model is simplified and does not contain the N-H and C=O atoms that are usually used to determine secondary structure, it was necessary to use a program capable of assigning secondary structure based only on the coordinates of C_α_ atoms; a program meeting this requirement is P-SEA [92]. For each snapshot, each residue was designated as α-helix, β-sheet, or coil, and a comparison with P-SEA's designation of the native state structure was used to determine whether the residue had assumed the correct native secondary structure. Averaging the results over all 10,000 snapshots—each of which was correctly weighted at 278 K using the Ferrenberg-Swendsen histogram methods [90,91]—allowed the mean probability of each residue assuming its native secondary structure to be computed.

### Measurements of local tertiary structure in fragments.

In order to provide an indication of local tertiary structure formation in fragments, the 10,000 structural snapshots discussed above were analyzed as follows. In the same way that the folding coordinate Q describes the fraction of all native contacts formed in a given conformation of the protein, a residue-specific folding coordinate Q_res_ can be defined for each residue that describes the fraction of *its* native contacts formed: defined in this way, Q_res_, therefore, has lower and upper limits of 0 and 1, respectively, for each residue. Averaging Q_res_ values computed for all 10,000 snapshots—and again correctly weighting each snapshot at 278 K using the Ferrenberg-Swendsen histogram methods—allowed the mean Q_res_ value of each residue to be computed.

### Computing the kinetics of refolding.

To model refolding of the full-length proteins (i.e., from an unfolded state), two separate sets of BD simulations were conducted starting from (1) conformations generated by randomly setting the dihedral angles of the protein chain, subject to the requirement that no bad steric clashes were incorporated, and (2) conformations randomly selected from the free energy minimum of the unfolded state ensemble sampled at T_fold_. Simulations were continued until Q = 0.95 was attained, at which point it was assumed that the protein had successfully folded; in the case of SFVP, an additional requirement was that all native contacts of the final residue trp149 be fully formed since this residue is crucial for the autoproteolysis reaction that terminates cotranslational folding [54]. Refolding trajectories for SFVP were also monitored with independent measures of folding for the N-terminal domain (Q_N_), the C-terminal domain (Q_C_), and the interface region (Q_INT_). Q_N_ was defined using native contacts in which both residues were in the N-terminal domain (residues 1–64); Q_C_ was defined using native contacts in which both residues were in the C-terminal domain (residues 65–149), and Q_INT_ was defined using native contacts in which one residue was in the N-terminal domain and the other was in the C-terminal domain.

In order to obtain a meaningful sample of the simulated refolding events for each protein, a number of independent trajectories were computed, each starting from a different conformation: for all proteins, the number of computed trajectories was 100. The estimates of the folding times so obtained were used in part to determine the rate of simulated protein synthesis in coupled synthesis and folding simulations (see below).

### Modeling coupled synthesis and folding.

For all simulations of ribosome-mediated synthesis and folding, the structural model of the large ribosomal subunit solved by Ban et al. was used (pdb file 1FFK; [53]) and aligned such that the exit of the ribosomal tunnel was directed along the −*x* direction, with the peptidyltransferase active site located near the origin of the coordinate frame. The ribosomal proteins in this structure are present at the same C_α_ level of resolution used to model the nascent protein in the BD simulations. The RNA, however, is represented at true atomic resolution, so in order that the simulated resolution of the proteins and nucleic acids be approximately similar in the simulations, all but the following RNA atoms in the structure were deleted: P, C1′, C3′, and C5′ atoms of the sugar-phosphate backbone, the N3, C6, and N7 atoms of purine bases, and the N3 and C5 atoms of pyrimidine bases. In order to allow the nascent protein to fit within the peptidyltransferase active site without experiencing major steric clashes, two additional atoms were removed: C6 of Ade2103 and C5 of Cyt2644. Even with these changes, the density of pseudo-atoms within the RNA is still somewhat greater than that within the protein parts of the ribosome; this, however, was found to be necessary to prevent the nascent protein burrowing unrealistically through the center of RNA helices. Interactions between pseudo-atoms of the nascent protein and the ribosome were modeled with the same purely repulsive energy function outlined above (Equation 3) for describing non-native interactions within the protein; σ_ij_ was set to 4 Å and ɛ was set in all simulations to 0.6 kcal/mol. In order to investigate how folding behavior might be affected by increasing the level of confinement experienced by the nascent chain within the ribosome tunnel, a series of coupled synthesis–folding simulations was also performed in which σ_ij_ for protein–ribosome interactions was set to larger values; 30 independent simulations were performed for each of the following σ_ij_ values: 4.0 Å, 4.5 Å, 5.0 Å, 5.5 Å, and 6.0 Å (see Results). Although in most of the simulations reported here there were no favorable energetic interactions between atoms of the nascent protein and the ribosome, additional simulations were also conducted in which the combined attractive–repulsive energy function (Equation 2) was used in place of the purely repulsive energy function (see Results); σ_ij_ was set to 4 Å, and 30 independent simulations were performed for ɛ values of 0.1 and 0.2 kcal/mol.

The synthesis and release of nascent protein transcripts was modeled within the ribosomal subunit in the following way. Simulations were begun with the first four residues of the protein already synthesized in the peptidyltransferase site and arranged in a *trans*-orientation (such that they formed a pseudo-dihedral angle of 180°), pointed roughly in the direction of the tunnel's exit. Residue 5 of the protein was initially placed in a position displaced 3 Å in the +*x* direction into the peptidyltransferase site. At the start of the simulation, only residues 1 to 4 were allowed to move freely according to the dictates of the BD algorithm; over each of 4 million simulation steps, however, residues 4 and 5 were both subjected to small translations of 7.5 × 10^−7^ Å in the −*x* direction until residue 5 was positioned at the same *x*-coordinate occupied by residue 4 at the beginning of the simulation. When this point was reached, residue 5 was released and allowed to freely move during BD, and the series of small translations (this time applied to residues 5 and 6) repeated in order to gradually introduce residue 6. This process was repeated until all residues of the protein were introduced and released. For CI2, barnase, and SFVP, the number of coupled synthesis–folding simulations performed for each set of energy parameters was 50, 30, and 10, respectively.

### Ensuring compatibility of simulated timescales.

The computed folding times of proteins represented by structurally simplified Gō models are orders of magnitude shorter than the corresponding experimental folding times; despite this the *relative* rates of folding of various single-domain proteins (at their folding temperatures) have been shown to be in surprisingly close agreement with experiment [31]. The disparity between experimental and simulation timescales takes on added importance in the present context, however, since it is vital that the rates of amino acid synthesis and protein folding be realistically matched in the simulations. In particular, a major concern is that if the simulated rate of synthesis is too high relative to the simulated folding rate (or the rate at which relaxation of the structure occurs), amino acids might rapidly “bunch up” unrealistically at the site of their synthesis. A secondary concern (secondary in the sense that it is less likely to be encountered) is that if the simulated rate of synthesis is too low, the protein's ability to sample widely different alternative conformations during synthesis might be unrealistically high.

In an attempt to determine an appropriate rate for “synthesizing” residues in the simulations, the experimental translation rate was first compared to the experimental refolding rates of CI2 and barnase. Although in vivo rates of translation can vary considerably [64,65], they can average around 20 residues per second [93], which is similar to the experimentally measured refolding rates of both CI2 and barnase in water (50s^−1^ [39] and 13s^−1^ [42], respectively). The same proteins refold in the simulations with median folding times of approximately 280 ns and approximately 395 ns, respectively (see Results) which indicates that new amino acids should be introduced in the simulations at intervals of perhaps a few hundred nanoseconds; because of computational considerations, a value of 100 ns was chosen, which, as noted above, corresponds to 4 million simulation steps. Control simulations of CI2 without favorable Gō interactions (and without the ribosome) indicated that this rate was sufficiently slow that the protein's radius of gyration remained at or near its equilibrium value throughout synthesis (unpublished data). As a more explicit test of the effects of using alternative timescales of synthesis, sets of 50 and five simulations were performed in which new amino acids were introduced at intervals of 10 ns and 1,000 ns, respectively; the smaller number of trajectories performed with the longer timescale of synthesis reflects the much greater computational overhead associated with such simulations.

## Supporting Information

Figure S1 Folding Free Energy Surfaces for Barnase Fragments (A–F) Free energy surfaces for fragments comprising residues 23–109, 1–68, 1–79, 1–95, 1–105 and 1–109 (full length), respectively. Free energy (G) is shown on a continuous color scale from 0 (red) to +5 kcal/mol (white); symbols show the mean value of Q in 5-K intervals.
(7.9 MB PDF)

Figure S2 Heat Capacities of CI2 and Barnase Fragments (A) Plot of heat capacity versus temperature for CI2 fragments. (B) Same as (A), but for barnase.
(92 KB PPT)

Figure S3 Time-Evolution of Q_res_ in CI2 and Barnase in the Absence of the Ribosome (A) Mean values of the residue-specific Q_res_ value during coupled synthesis–folding of CI2 in the absence of the ribosome; separate lines are plotted for each residue, colored from blue to red. (B) Same as (A), but for barnase; note that for clarity, only the period of synthesis of the first 60 residues is shown.
(120 KB PPT)

Figure S4 Trapping of Artificially Stabilized CI2 in the Ribosome Tunnel Snapshots from a coupled synthesis–folding simulation of CI2 performed with the artificially exaggerated energy parameter (ɛ = 0.80 kcal/mol); note that the protein becomes stuck for approximately 4 μs in partially folded conformations.
(7.2 MB PPT)

Figure S5 Escape of Trapped CI2 When a Realistic Energy Parameter Is Used Snapshots from a restarted “stuck” CI2 simulation in which the artificially exaggerated energy parameter (ɛ = 0.80 kcal/mol) has been replaced by the realistic parameter (ɛ = 0.60 kcal/mol); the protein rapidly loses tertiary structure, exits the tunnel, and completes folding in solution.
(3.7 MB PPT)

Figure S6 Parameter Dependence of Folding Trajectories for SFVP Plots of Q versus time for ten independent coupled synthesis–folding simulations of SFVP in the presence of the ribosome. (A–C) Results obtained with three different energy parameters.
(572 KB PPT)

Figure S7 A Typical Refolding Trajectory for SFVP Snapshots from a typical refolding trajectory of SFVP computed with ɛ = 0.54 kcal/mol. The protein is colored from blue (N-terminus) to red (C-terminus); note that the two domains fold independently first, with association of the two domains constituting a final step.
(1.2 MB PPT)

Figure S8 Coupled Synthesis–Folding Trajectories for SFVP with ɛ = 0.54 Trajectories of ten independent coupled synthesis–folding simulations of SFVP in the presence of the ribosome with ɛ = 0.54 kcal/mol. Note that folding of the N-terminal domain usually precedes the later folding and association of the C-terminal domain.
(995 KB PPT)

Figure S9 Coupled Synthesis–Folding Trajectories for SFVP with ɛ = 0.57 Trajectories of ten independent coupled synthesis–folding simulations of SFVP in the presence of the ribosome with ɛ = 0.57 kcal/mol. Note that folding of the C-terminal domain occurs in concert with its association with the N-terminal domain.
(906 KB PPT)

Figure S10 Refolding and De Novo Folding of SFVP with ɛ = 0.57 kcal/mol (A) The most populated points in (Q_N_, Q_C_, Q_INT_) space during 100 refolding trajectories. Each point in the (Q_N_, Q_C_, Q_INT_) space is counted only once per trajectory in order to prevent repeated revisiting of the same region in a trajectory from biasing the results. Symbol size and color reflect relative population (large, red symbols being populated in 100% of simulations). (B) Same as (A), but for ten coupled synthesis–folding simulations.
(370 KB PPT)

Figure S11 Effect of Altering the Interaction between the Nascent Protein and the Ribosome on Q in Coupled Synthesis–Folding Simulations of CI2 (A) Mean values of Q during coupled synthesis–folding of CI2 in the presence (filled symbols) and absence (open symbols) of the ribosome obtained using σ_ij_ = 4.0 Å for protein–ribosome interactions; error bars indicate the standard deviation of values obtained from 30 independent trajectories. (B) Same as (A), but filled symbols indicate results obtained with σ_ij_ = 4.5 Å; open symbols again refer to de novo folding in the absence of the ribosome. (C) Same as (B), but with σ_ij_ = 5.0 Å. (D) Same as (B), but with σ_ij_ = 5.5 Å. (E) Same as (B), but with σ_ij_ = 6.0 Å. (F) Same as (B), but with σ_ij_ = 4.0 Å and an attractive potential of well depth ɛ = 0.1 kcal/mol. (G) Same as (F), but with an attractive potential of well depth ɛ = 0.2 kcal/mol.
(165 KB PPT)

Figure S12 Effect of Altering the Interaction between the Nascent Protein and the Ribosome on the De Novo Folding Mechanism in Coupled Synthesis–Folding Simulations of CI2 (A) Plot of Q_res_ versus Q for the post-synthesis stage of de novo folding of CI2 obtained with σ_ij_ = 4.0 Å. (B) Same as (A), but with σ_ij_ = 4.5 Å. (C) Same as (A), but with σ_ij_ = 5.0 Å. (D) Same as (A), but with σ_ij_ = 5.5 Å. (E) Same as (A), but with σ_ij_ = 6.0 Å. (F) Same as (A), but with σ_ij_ = 4.0 Å and an attractive potential of well depth ɛ = 0.1 kcal/mol. (G) Same as (F), but with an attractive potential of well depth ɛ = 0.2 kcal/mol.
(1.2 MB PPT)

Figure S13 Effect of Altering the Balance between Local and Non-local Interactions on Q in Coupled Synthesis–Folding Simulations of CI2 (A) Plot of free energy, G, versus Q at 300 K full-length CI2 computed with the four parameter sets investigated (see text). (B) Plot of heat capacity versus temperature for full-length CI2 computed with each parameter set. (C) Mean values of Q during coupled synthesis–folding of CI2 in the presence (filled symbols) and absence (open symbols) of the ribosome with V_1_ = 0.125 kcal/mol; error bars indicate the standard deviation of values obtained from 30 independent trajectories. (D) Same as (C), but with V_1_ = 0.25 kcal/mol. (E) Same as (C), but with V_1_ = 0.5 kcal/mol. (F) Same as (C), but with V_1_ = 1.0 kcal/mol.
(139 KB PPT)

Figure S14 Correspondence between P_fold_ and the Free Energy G for CI2 and Barnase (A) Plot showing the free energy, G, and P_fold_ as a function of Q for CI2; error bars for the P_fold_ plot represent the standard deviation of values calculated for 500 sampled structures in each Q bin. (B) Same as (A), but for barnase.
(54 KB PPT)

Text S1 Discussion of Limitations of Gō-Type Models for Protein Folding (92 KB DOC)

## Abbreviations

BD: Brownian dynamics
CI2: chymotrypsin inhibitor 2
G: free energy
P_fold_: probability of reaching the folded state
Q: folding (reaction) coordinate
QC: Q for the C-terminal domain
Q_INT_: Q for the interface region
Q_N_: Q for the N-terminal domain
Q_res_: residue-specific folding reaction coordinate
SFVP: Semliki forest virus protein
T_fold_: folding temperature
ΔG°_fold_: free energy of folding
ɛ: energy well depth
