# Supplementary figures and images for: Molecular Simulations of Cotranslational Protein Folding: Fragment Stabilities, Folding Cooperativity, and Trapping in the Ribosome
Source: PLoS Comput Biol. 2006 Jul 28;2(7):e98. doi: 10.1371/journal.pcbi.0020098 (PMC1523309; doi:10.1371/journal.pcbi.0020098)

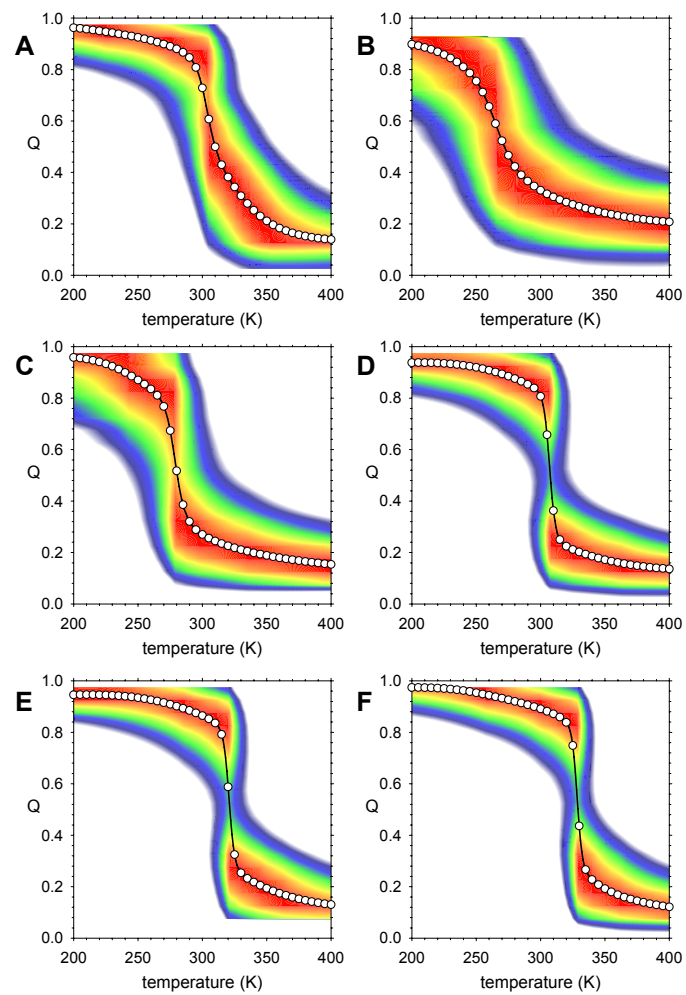

Figure S1

Supplement: Figure S1 — (A–F) Free energy surfaces for fragments comprising residues 23–109, 1–68, 1–79, 1–95, 1–105 and 1–109 (full length), respectively. Free energy (G) is shown on a continuous color scale from 0 (red) to +5 kcal/mol (white); symbols show the mean value of Q in 5-K intervals. (7.9 MB PDF) [file pcbi.0020098.sg001.pdf]

## Slide 1
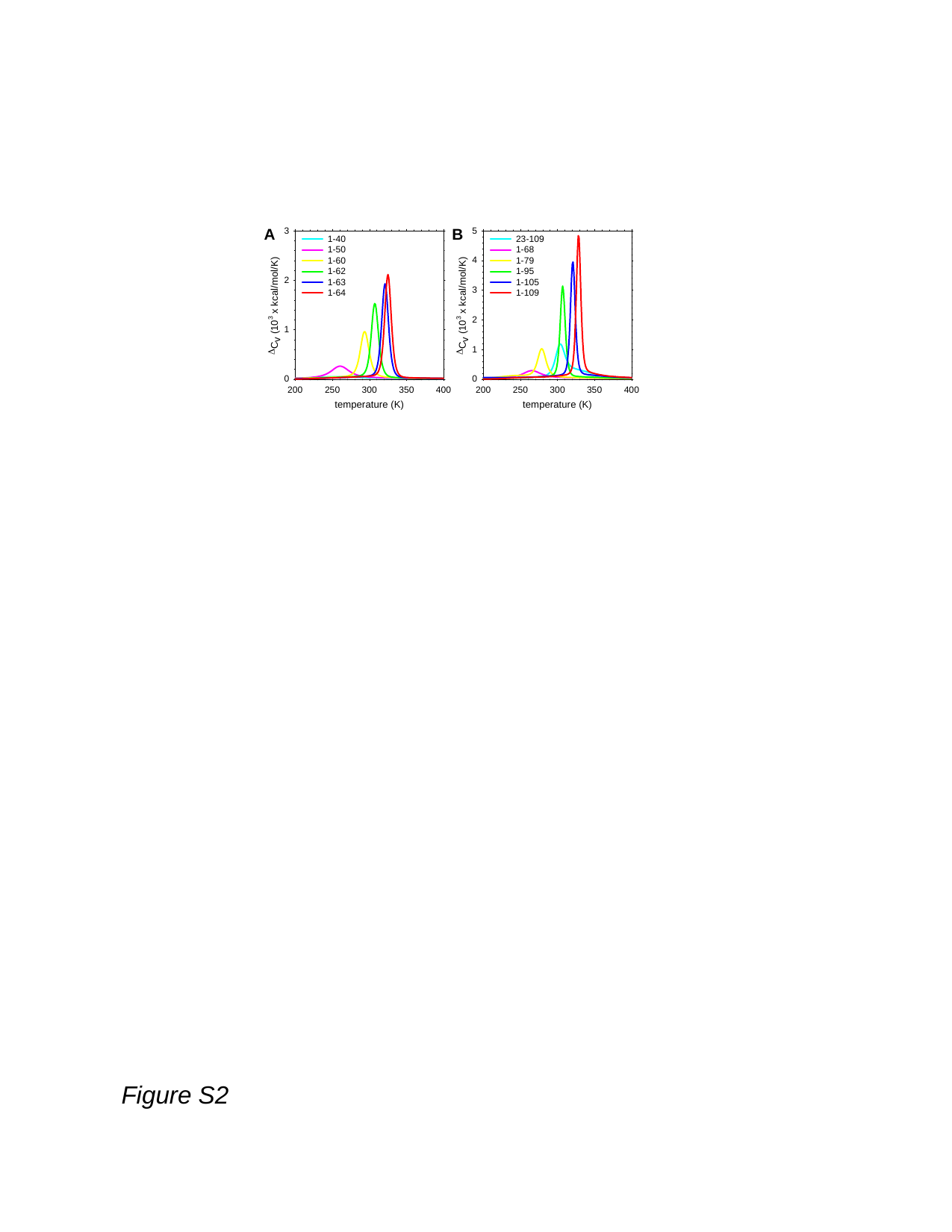

A
 B
Figure S2

Supplement: Figure S2 — (A) Plot of heat capacity versus temperature for CI2 fragments. (B) Same as (A), but for barnase. (92 KB PPT) [file pcbi.0020098.sg002.ppt]

## Slide 1
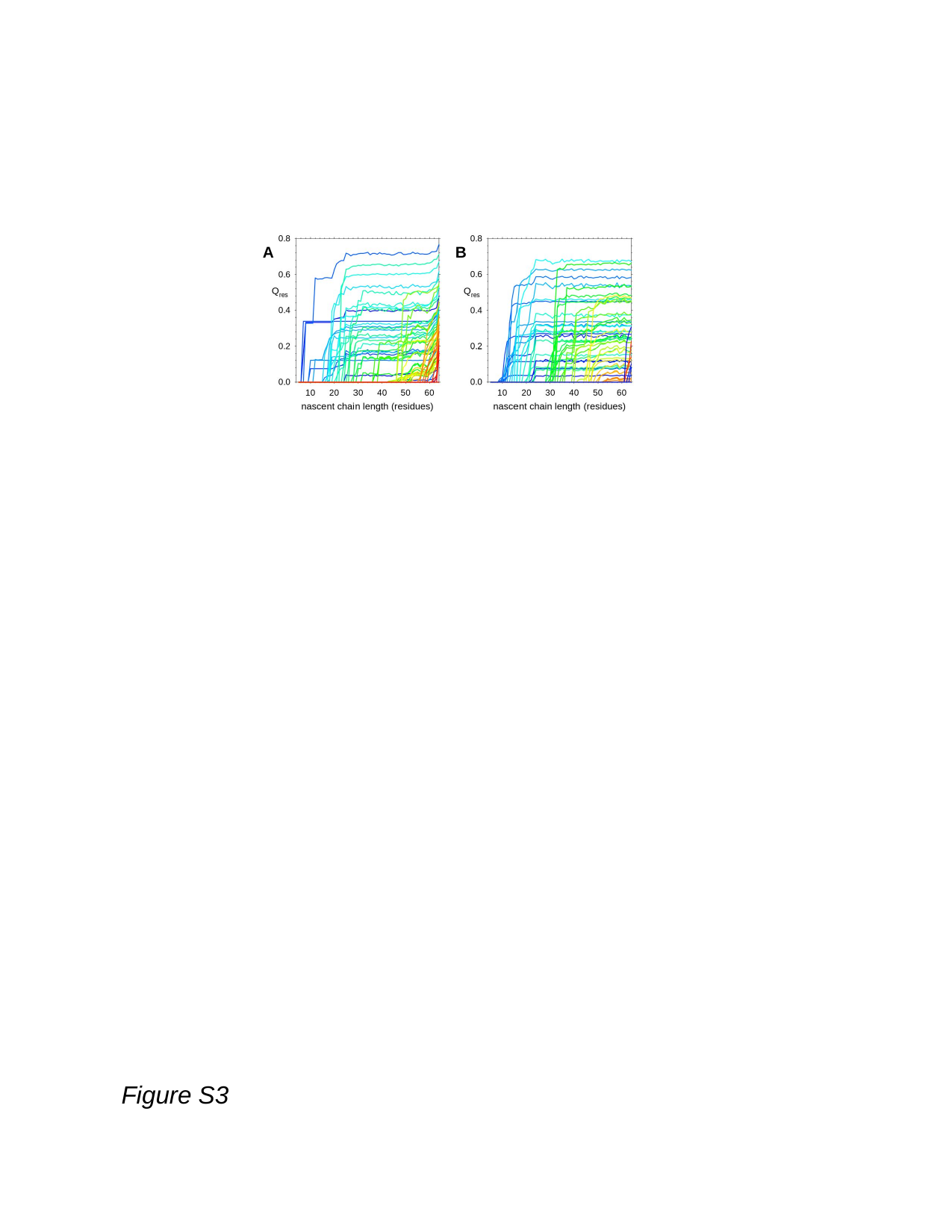

A
B
Figure S3

Supplement: Figure S3 — (A) Mean values of the residue-specific Qres value during coupled synthesis–folding of CI2 in the absence of the ribosome; separate lines are plotted for each residue, colored from blue to red. (B) Same as (A), but for barnase; note that for clarity, only the period of synthesis of the first 60 residues is shown. (120 KB PPT) [file pcbi.0020098.sg003.ppt]

## Slide 1
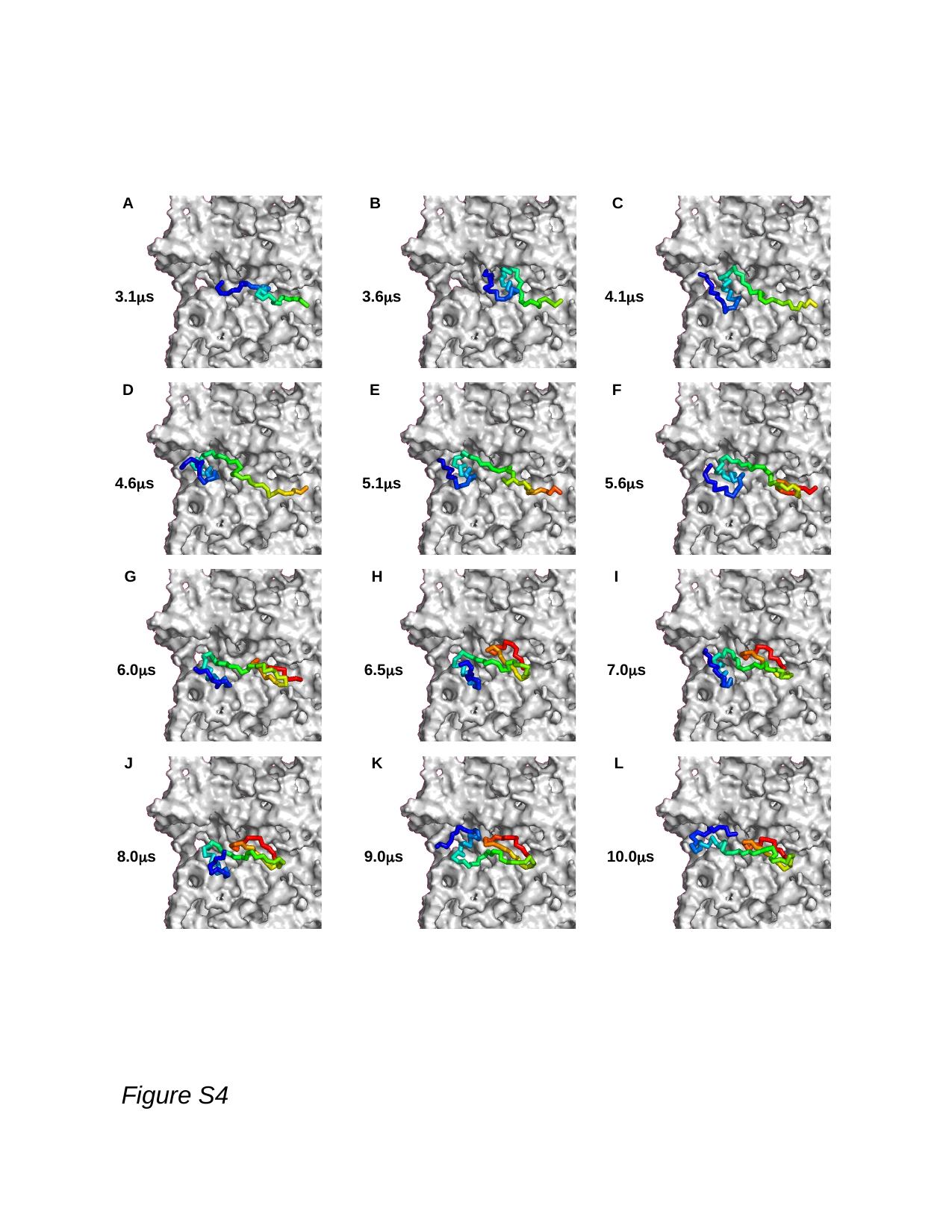

A
B
C
3.1μs
3.6μs
4.1μs
D
E
F
4.6μs
5.1μs
5.6μs
G
H
I
6.0μs
6.5μs
7.0μs
J
K
L
8.0μs
9.0μs
10.0μs
Figure S4

Supplement: Figure S4 — Snapshots from a coupled synthesis–folding simulation of CI2 performed with the artificially exaggerated energy parameter (ɛ = 0.80 kcal/mol); note that the protein becomes stuck for approximately 4 μs in partially folded conformations. (7.2 MB PPT) [file pcbi.0020098.sg004.ppt]

## Slide 1
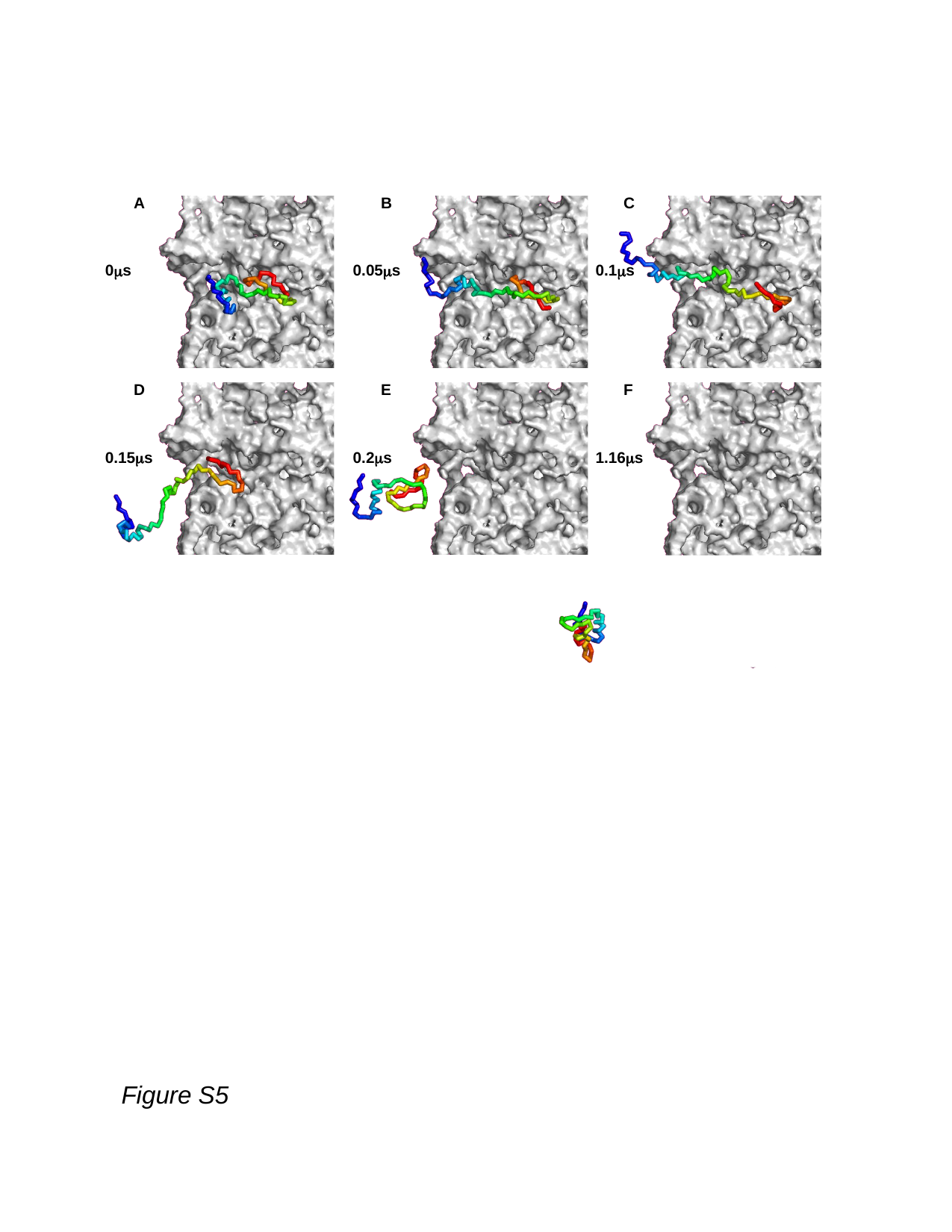

A
B
C
0μs
0.05μs
0.1μs
D
E
F
0.15μs
0.2μs
1.16μs
Figure S5

Supplement: Figure S5 — Snapshots from a restarted “stuck” CI2 simulation in which the artificially exaggerated energy parameter (ɛ = 0.80 kcal/mol) has been replaced by the realistic parameter (ɛ = 0.60 kcal/mol); the protein rapidly loses tertiary structure, exits the tunnel, and completes folding in solution. (3.7 MB PPT) [file pcbi.0020098.sg005.ppt]

## Slide 1
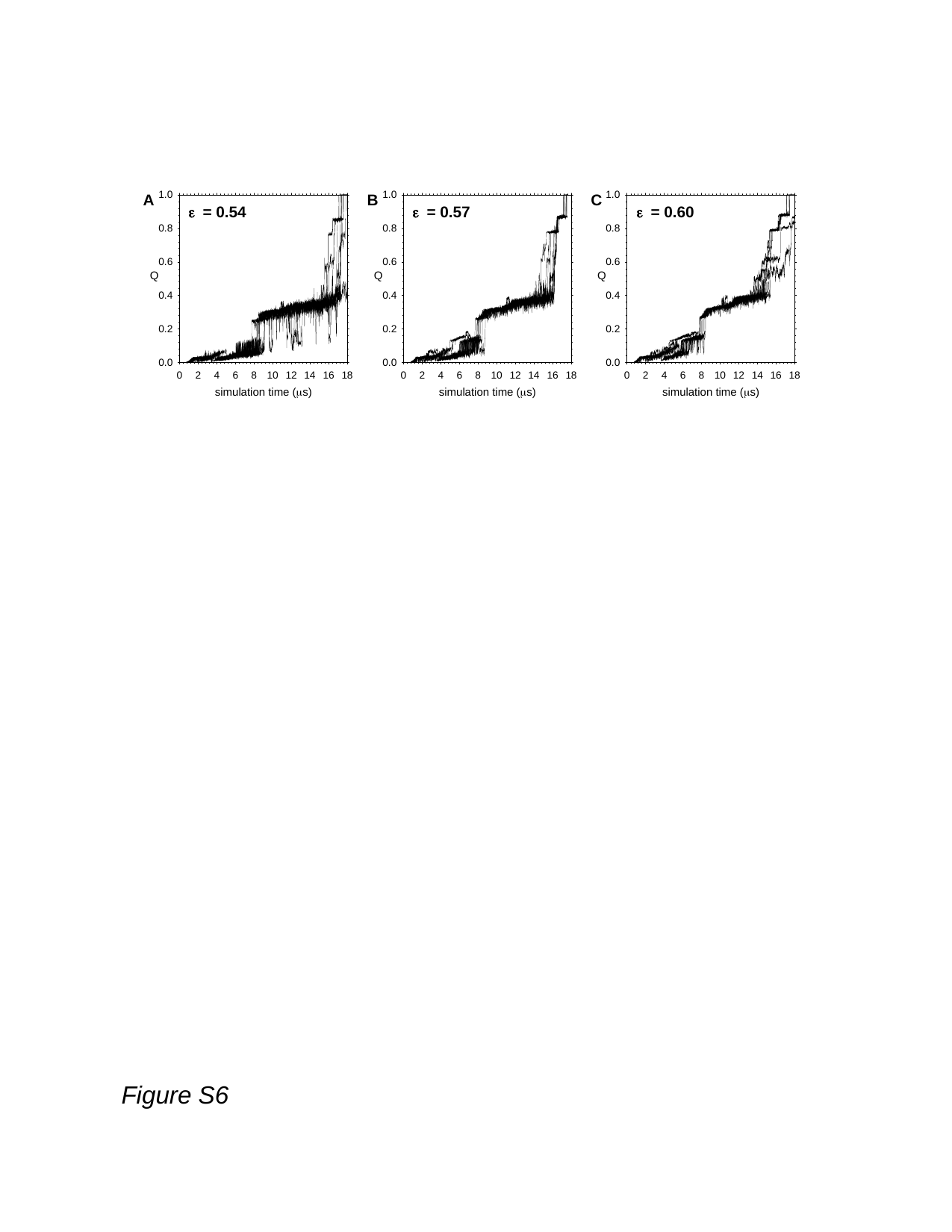

A
B
C
ε = 0.54
ε = 0.57
ε = 0.60
Figure S6

Supplement: Figure S6 — Plots of Q versus time for ten independent coupled synthesis–folding simulations of SFVP in the presence of the ribosome. (A–C) Results obtained with three different energy parameters. (572 KB PPT) [file pcbi.0020098.sg006.ppt]

## Slide 1
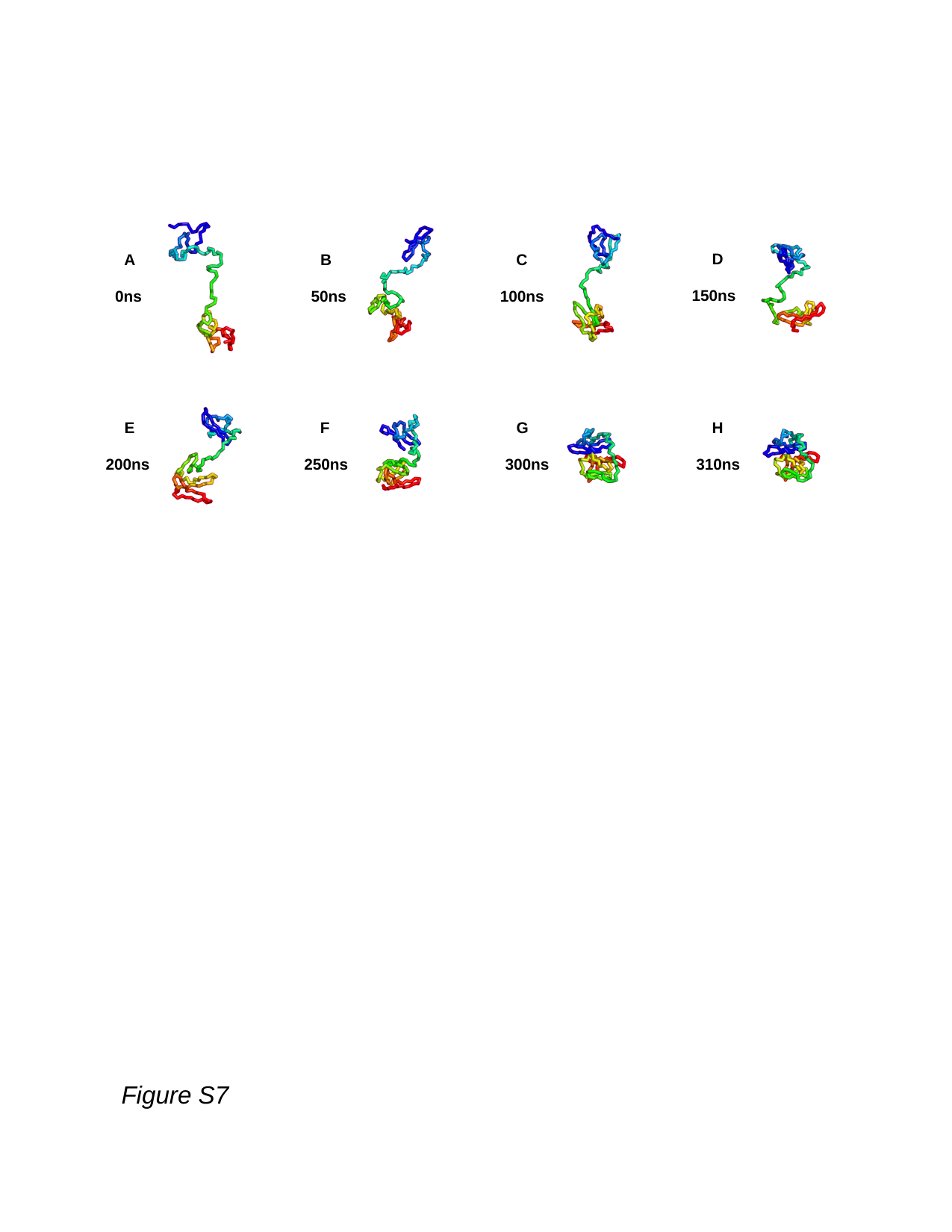

D
A
B
C
150ns
0ns
50ns
100ns
E
F
G
H
200ns
250ns
300ns
310ns
Figure S7

Supplement: Figure S7 — Snapshots from a typical refolding trajectory of SFVP computed with ɛ = 0.54 kcal/mol. The protein is colored from blue (N-terminus) to red (C-terminus); note that the two domains fold independently first, with association of the two domains constituting a final step. (1.2 MB PPT) [file pcbi.0020098.sg007.ppt]

## Slide 1
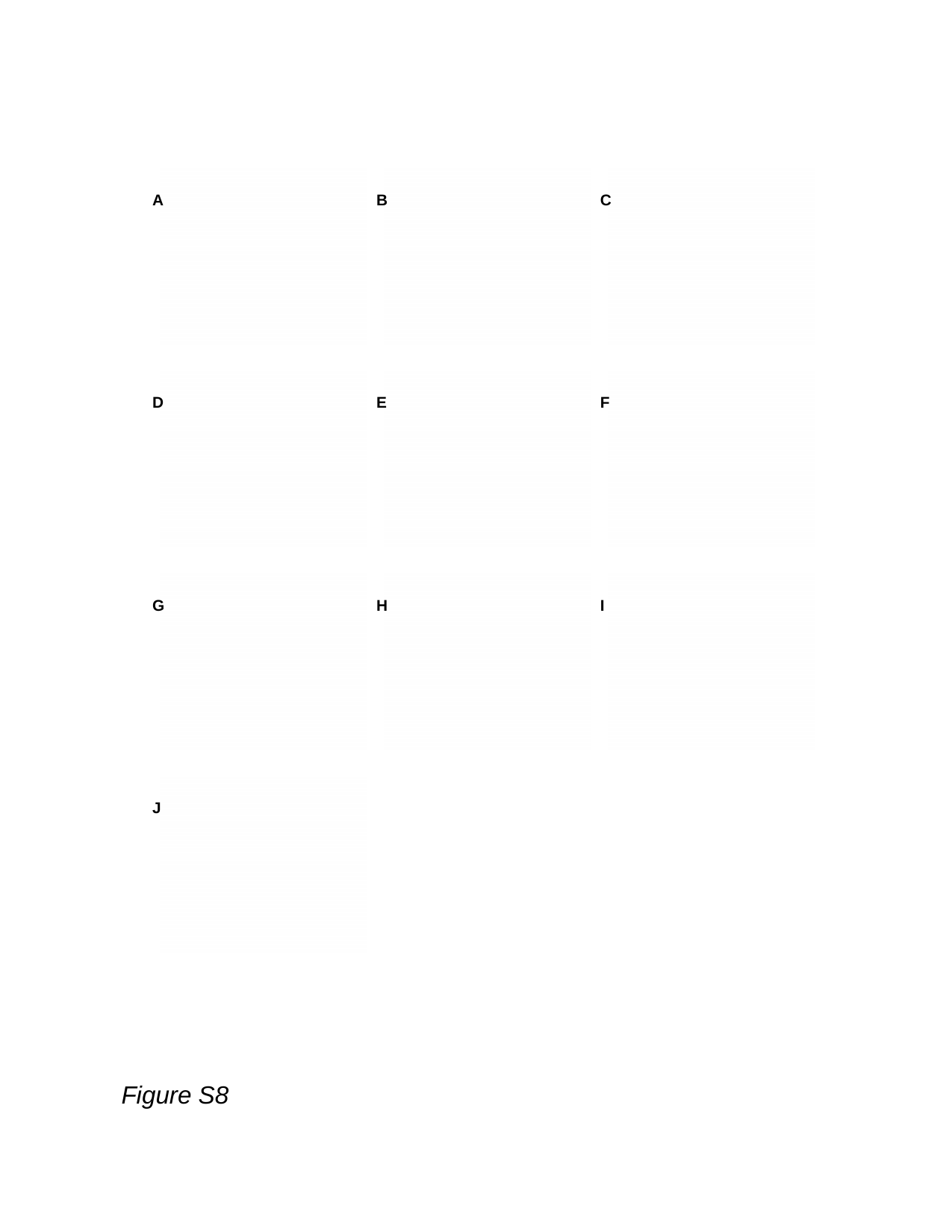

A
B
C
D
E
F
G
H
I
J
Figure S8

Supplement: Figure S8 — Trajectories of ten independent coupled synthesis–folding simulations of SFVP in the presence of the ribosome with ɛ = 0.54 kcal/mol. Note that folding of the N-terminal domain usually precedes the later folding and association of the C-terminal domain. (995 KB PPT) [file pcbi.0020098.sg008.ppt]

## Slide 1
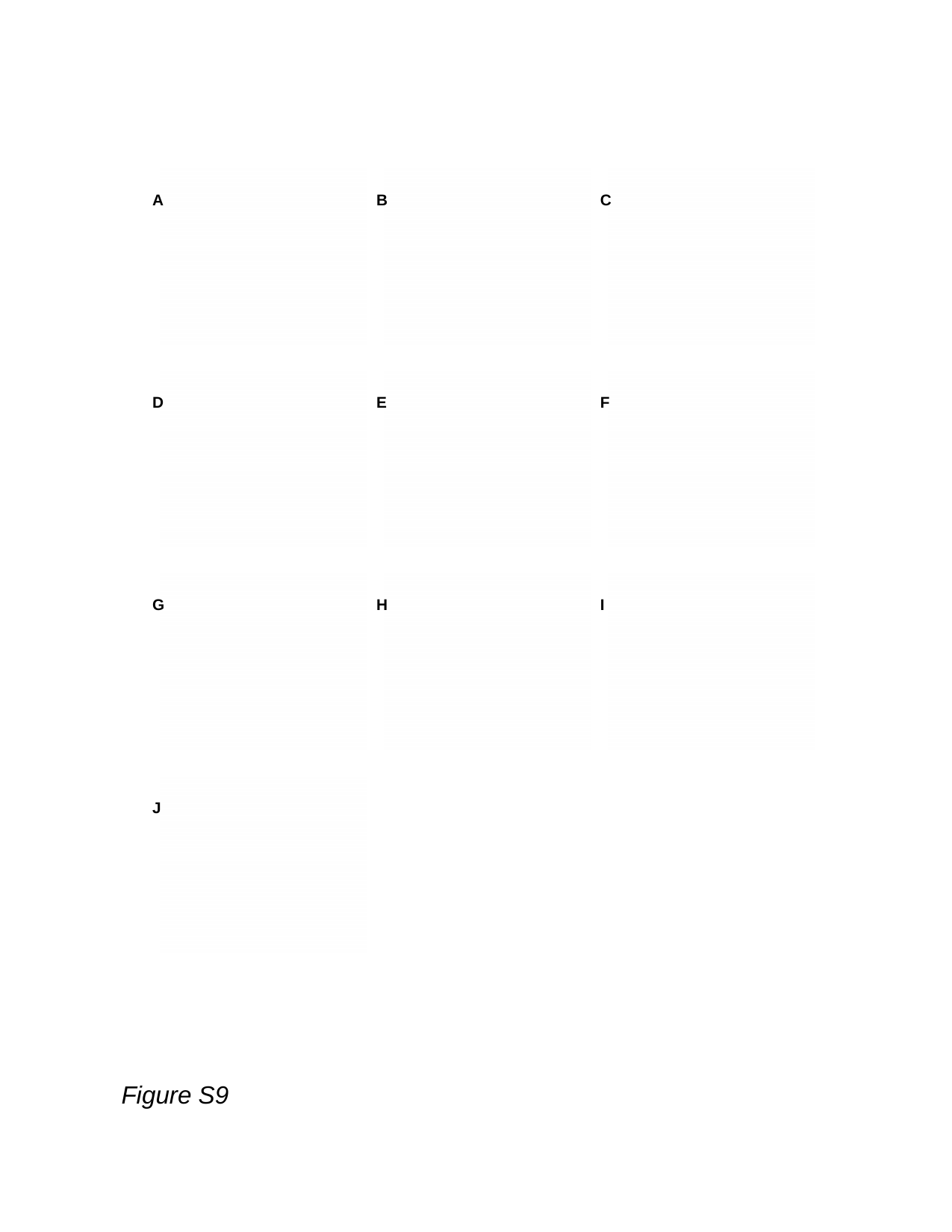

A
B
C
D
E
F
G
H
I
J
Figure S9

Supplement: Figure S9 — Trajectories of ten independent coupled synthesis–folding simulations of SFVP in the presence of the ribosome with ɛ = 0.57 kcal/mol. Note that folding of the C-terminal domain occurs in concert with its association with the N-terminal domain. (906 KB PPT) [file pcbi.0020098.sg009.ppt]

## Slide 1
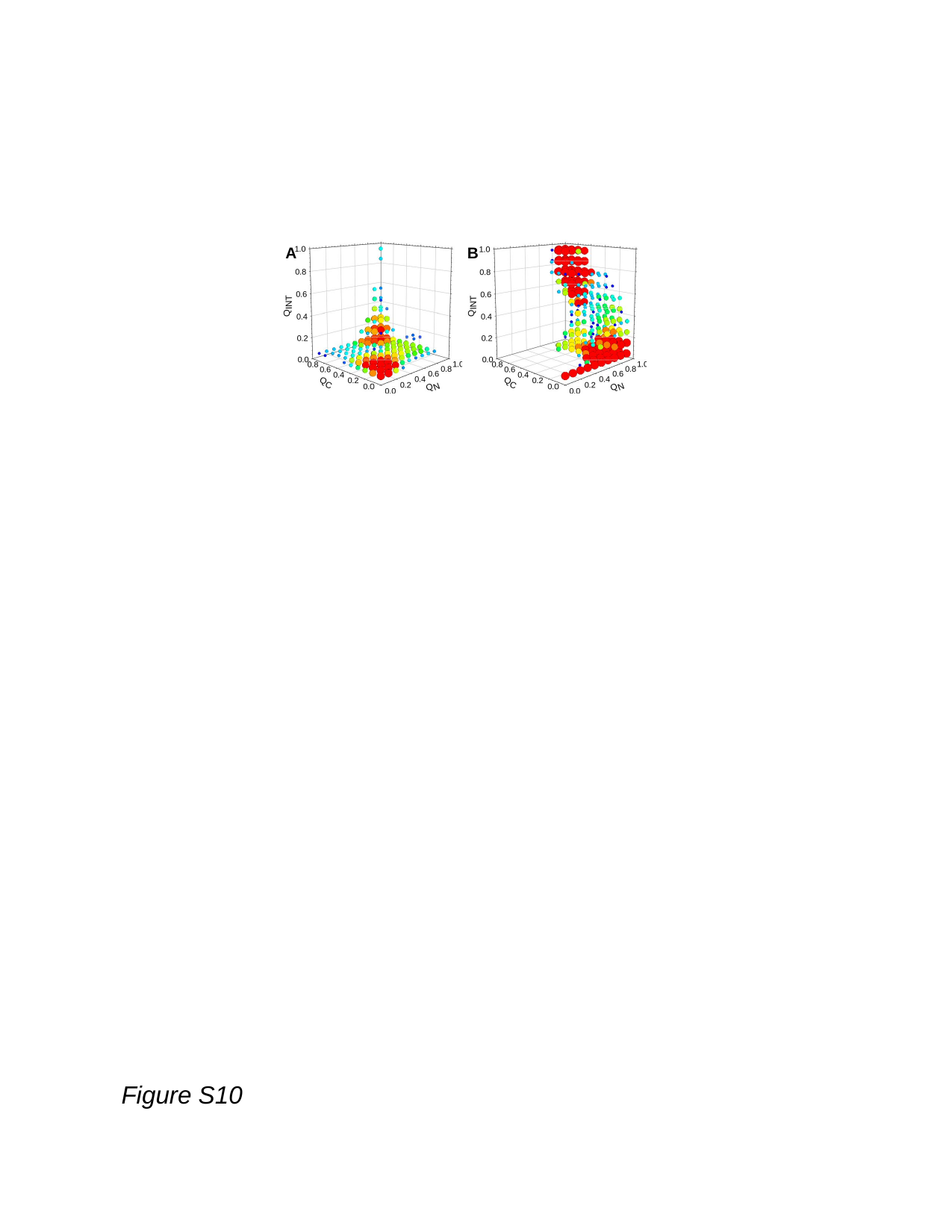

A
B
Figure S10

Supplement: Figure S10 — (A) The most populated points in (QN, QC, QINT) space during 100 refolding trajectories. Each point in the (QN, QC, QINT) space is counted only once per trajectory in order to prevent repeated revisiting of the same region in a trajectory from biasing the results. Symbol size and color reflect relative population (large, red symbols being populated in 100% of simulations). (B) Same as (A), but for ten coupled synthesis–folding simulations. (370 KB PPT) [file pcbi.0020098.sg010.ppt]

## Slide 1
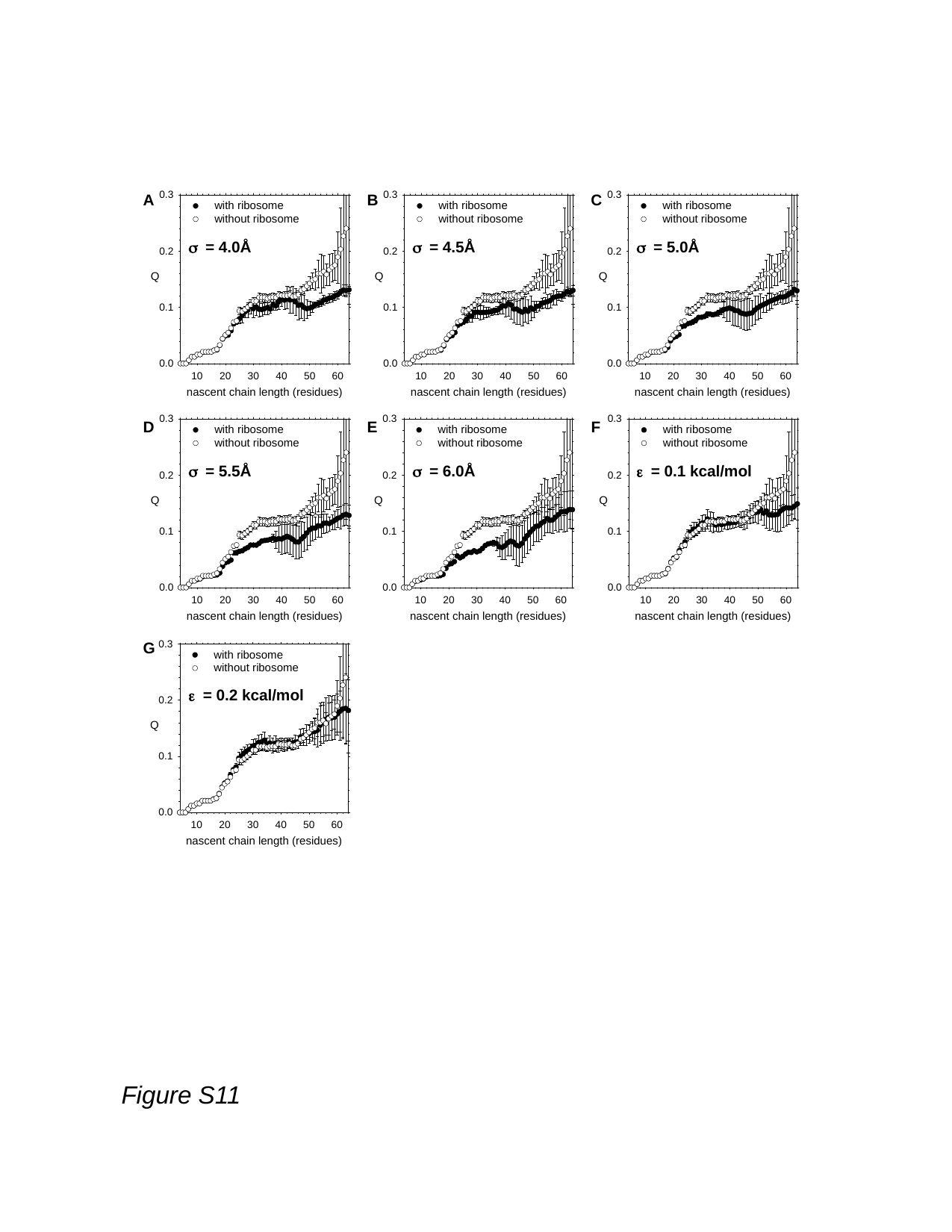

A
B
C
 = 4.0Å
 = 4.5Å
 = 5.0Å
D
E
F
 = 5.5Å
 = 6.0Å
ε = 0.1 kcal/mol
G
ε = 0.2 kcal/mol
Figure S11

Supplement: Figure S11 — (A) Mean values of Q during coupled synthesis–folding of CI2 in the presence (filled symbols) and absence (open symbols) of the ribosome obtained using σij = 4.0 Å for protein–ribosome interactions; error bars indicate the standard deviation of values obtained from 30 independent trajectories. (B) Same as (A), but filled symbols indicate results obtained with σij = 4.5 Å; open symbols again refer to de novo folding in the absence of the ribosome. (C) Same as (B), but with σij = 5.0 Å. (D) Same as (B), but with σij = 5.5 Å. (E) Same as (B), but with σij = 6.0 Å. (F) Same as (B), but with σij = 4.0 Å and an attractive potential of well depth ɛ = 0.1 kcal/mol. (G) Same as (F), but with an attractive potential of well depth ɛ = 0.2 kcal/mol. (165 KB PPT) [file pcbi.0020098.sg011.ppt]

## Slide 1
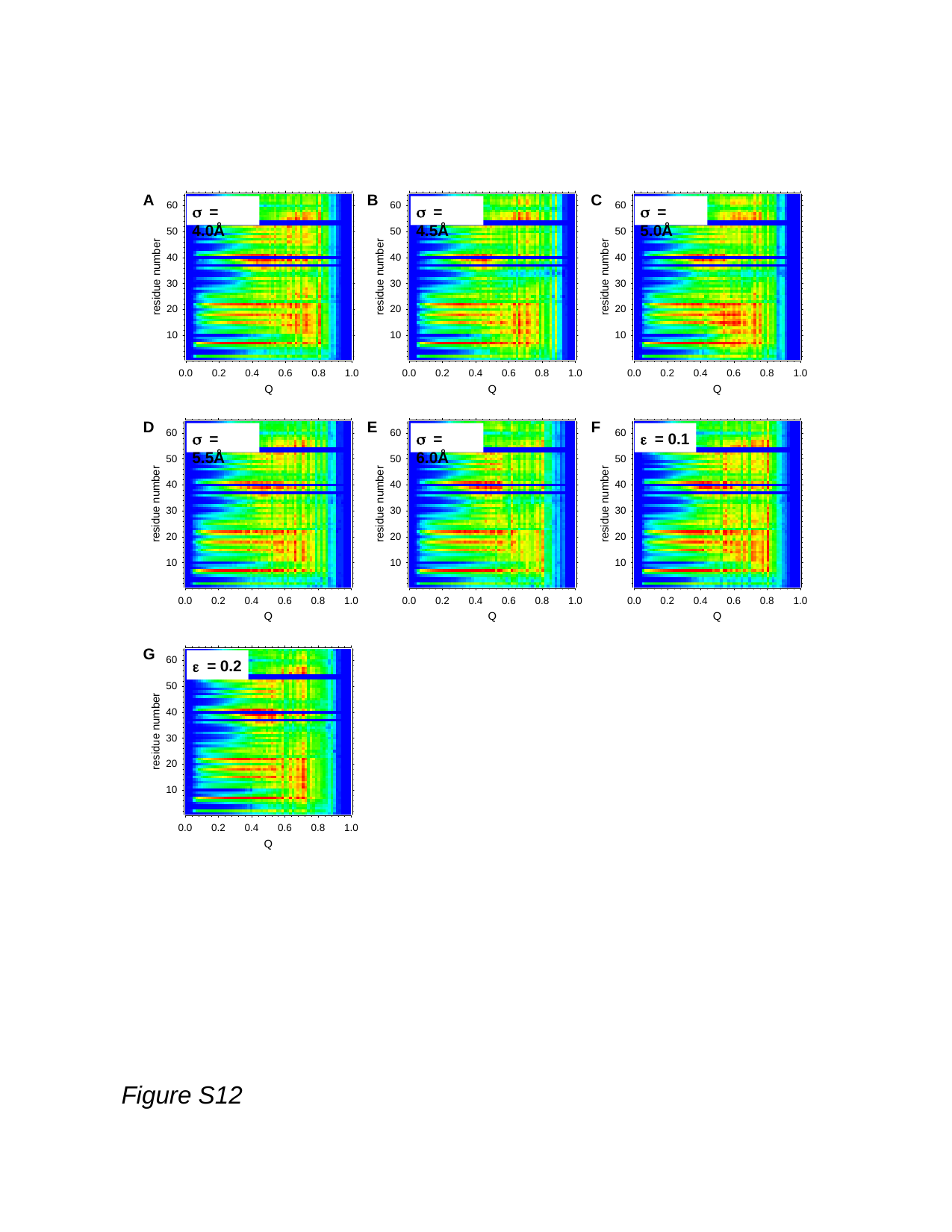

A
B
C
 = 4.0Å
 = 4.5Å
 = 5.0Å
D
E
F
 = 5.5Å
 = 6.0Å
ε = 0.1
G
ε = 0.2
Figure S12

Supplement: Figure S12 — (A) Plot of Qres versus Q for the post-synthesis stage of de novo folding of CI2 obtained with σij = 4.0 Å. (B) Same as (A), but with σij = 4.5 Å. (C) Same as (A), but with σij = 5.0 Å. (D) Same as (A), but with σij = 5.5 Å. (E) Same as (A), but with σij = 6.0 Å. (F) Same as (A), but with σij = 4.0 Å and an attractive potential of well depth ɛ = 0.1 kcal/mol. (G) Same as (F), but with an attractive potential of well depth ɛ = 0.2 kcal/mol. (1.2 MB PPT) [file pcbi.0020098.sg012.ppt]

## Slide 1
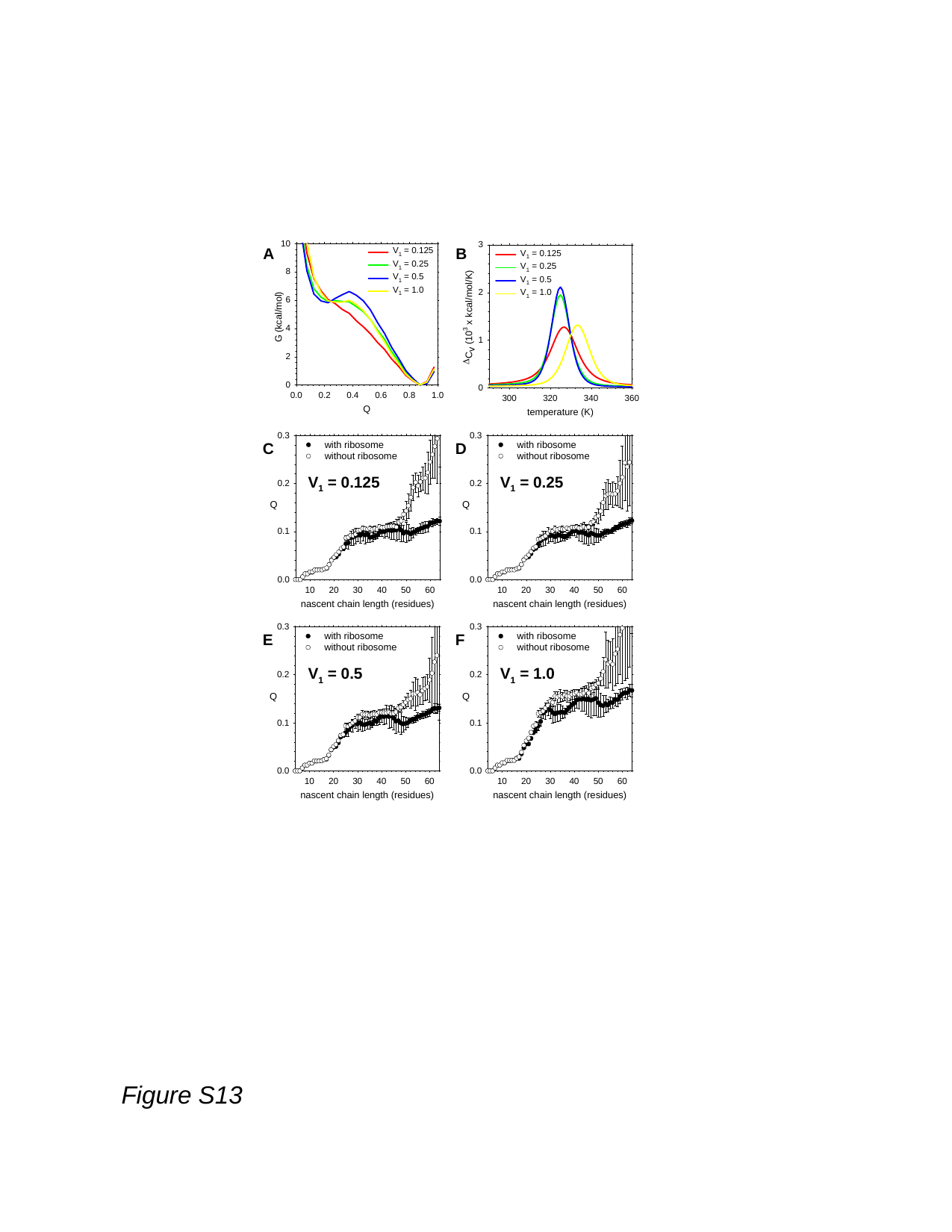

A
B
C
D
V1 = 0.125
V1 = 0.25
E
F
V1 = 0.5
V1 = 1.0
Figure S13

Supplement: Figure S13 — (A) Plot of free energy, G, versus Q at 300 K full-length CI2 computed with the four parameter sets investigated (see text). (B) Plot of heat capacity versus temperature for full-length CI2 computed with each parameter set. (C) Mean values of Q during coupled synthesis–folding of CI2 in the presence (filled symbols) and absence (open symbols) of the ribosome with V1 = 0.125 kcal/mol; error bars indicate the standard deviation of values obtained from 30 independent trajectories. (D) Same as (C), but with V1 = 0.25 kcal/mol. (E) Same as (C), but with V1 = 0.5 kcal/mol. (F) Same as (C), but with V1 = 1.0 kcal/mol. (139 KB PPT) [file pcbi.0020098.sg013.ppt]

## Slide 1
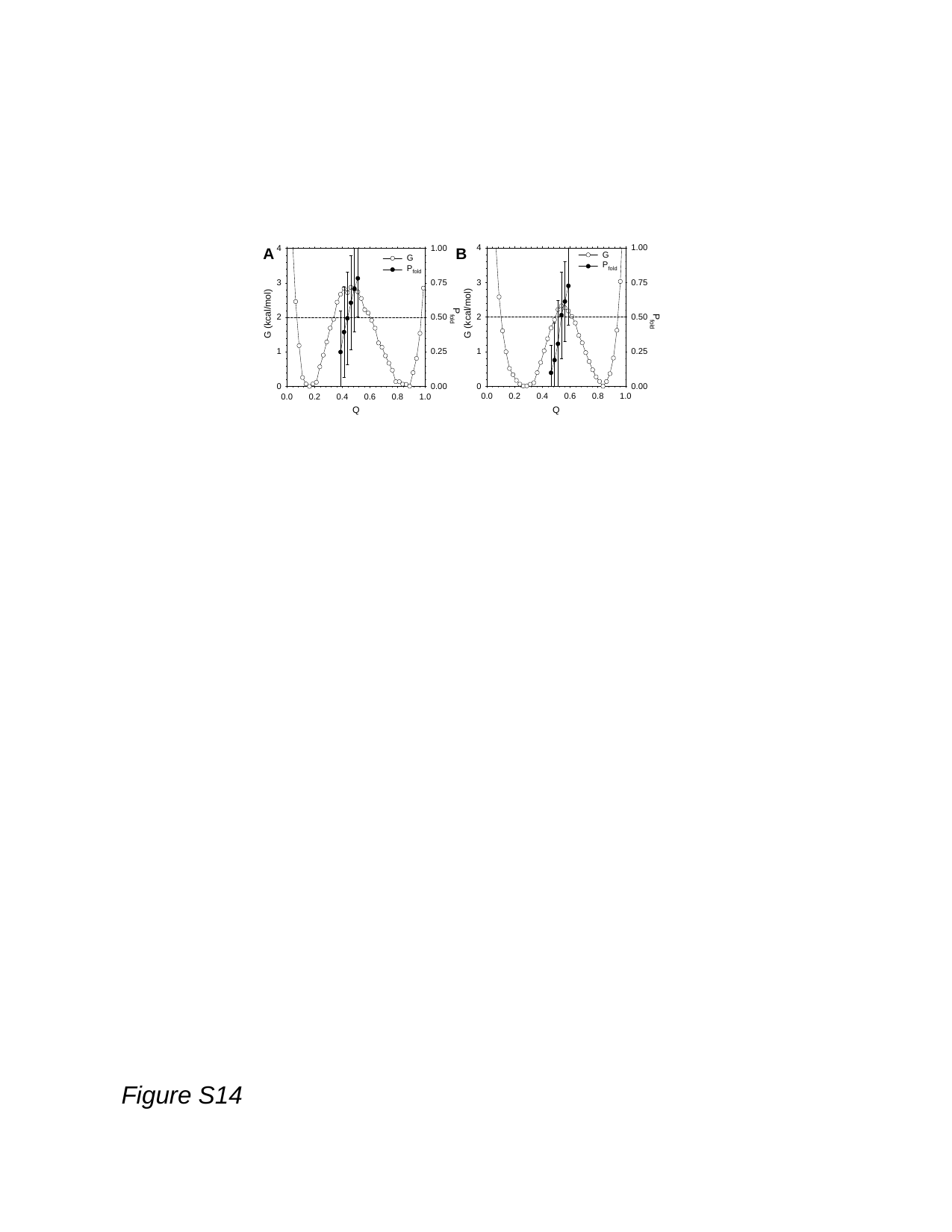

A
B
Figure S14

Supplement: Figure S14 — (A) Plot showing the free energy, G, and Pfold as a function of Q for CI2; error bars for the Pfold plot represent the standard deviation of values calculated for 500 sampled structures in each Q bin. (B) Same as (A), but for barnase. (54 KB PPT) [file pcbi.0020098.sg014.ppt]
