# Supplementary material for: Molecular Simulations of Cotranslational Protein Folding: Fragment Stabilities, Folding Cooperativity, and Trapping in the Ribosome
Source: PLoS Comput Biol. 2006 Jul 28;2(7):e98. doi: 10.1371/journal.pcbi.0020098 (PMC1523309; doi:10.1371/journal.pcbi.0020098)
Supplement: Text S1 — (92 KB DOC) [file pcbi.0020098.sd001.doc]

*Limitations of the Gō methodology*

One key assumption of the present work is that a simple simulation model developed to study protein *refolding* events can be extended and applied to model the more complex phenomenon of coupled synthesis and folding within the large ribosomal subunit. Because this assumption is a critical one, it is important to review the advantages and disadvantages of the folding model. In the present context, the major advantage of the protein folding model used here is its computational simplicity: in fact, it is the combination of a residue-level structural description and a Gō-type [1,2] description of the residue-residue interactions that makes the simulations discussed here feasible with modest computational resources. Importantly, previous studies have indicated that the apparently outrageous simplicity of the model does not prevent it from successfully describing some of the key features of refolding events [2-6], and the same basic model has also recently found success in describing coupled folding and binding reactions [7,8].

Not surprisingly however, the model does have known drawbacks, and one that is reiterated here is that the details of its computed folding mechanisms are not always in complete agreement with experiment [3,4], nor with the more detailed views obtained from all-atom molecular dynamics simulations [9]. Attempts have been made to develop more sophisticated Gō-type models (e.g. to allow favorable non-native interactions) while retaining the simplicity that makes them computationally attractive [10-12]; it has for example been shown recently that the energies of residue-residue contacts can be adjusted individually to more closely mould the simulated transition state ensemble to agree with experiment [12]. A second documented drawback of Gō-type models is that when used to investigate the dependence of refolding kinetics on the strength of native contacts, they can exhibit an excessive ‘chevron roll-over’, apparently as a result of partially folded structures forming kinetic traps [13,14]; this effect may well be the cause of the slight bimodality of CI2 refolding times obtained with ε = 0.80 kcal/mol (Figure 8A). A third known issue is that the simulated folding temperatures (T_f_) in Gō-type models appear to show a spurious dependence on the number of native contacts (M) and the number of residues in the protein (N), such that T_f_ ~ M/N [5]; the causes of this are yet to be examined in detail, but may be partly due to the neglect of the heat capacity effects of energetic interactions. It has been shown that when corrected for these artifactual differences in folding temperatures, the simulated folding rates of single-domain proteins obtained with Gō-like models span the same *ten* orders of magnitude range as the corresponding experimental folding rates [5].

A reviewer raised the question of whether the (artifactual) dependence of folding temperature on the number of native contacts might make the Gō model’s reproduction of fragment stabilities actually a trivial result. As shown graphically below, it turns out in fact that the ratio M/N *is* a rather good – though not perfect – predictor of the simulated stability of the various fragments at 300K. That said however M/N does not appear to provide any indication of how the cooperativity of the thermal unfolding transition will depend on fragment length (see Figure 2).

CI2

barnase

*References*

1. Gō N (1983) Theoretical-studies of protein folding. Annu Rev Biophys Bioeng 12:183-210.
2. Takada S (1999) Gōing for the prediction of protein folding mechanisms. Proc Natl Acad Sci USA 96:11698-11700.
3. Clementi C, Nymeyer H, Onuchic JN (2000) Topological and energetic factors: what determines the structural details of the transition state ensemble and “en-route” intermediates for protein folding? An investigation for small globular proteins. J Mol Biol 298:937-953.
4. Koga N, Takada S (2001) Role of native topology and chain-length scaling in protein folding: a simulation study with a Go-like model. J Mol Biol 313:171-180.
5. Chavez LL, Onuchic JN, Clementi C (2004) Quantifying the roughness on the free energy landscape: entropic bottlenecks and protein folding rates. J Am Chem Soc 126:8426-8432.
6. Das P, Wilson CJ, Fossati G, Wittung-Stafshede P, Matthews KS, Clementi C (2005) Characterization of the folding landscape of monomeric lactose repressor: quantitative comparison of theory and experiment. Proc Natl Acad Sci USA 102:14569-14574.
7. Levy Y, Wolynes PG, Onuchic JN (2004) Protein topology determines binding mechanism. Proc Natl Acad Sci USA 101:511-516.
8. Levy Y, Cho SS, Onuchic JN, Wolynes PG (2005) A survey flexible protein binding mechanisms and their transition states using native topology based energy landscapes. J Mol Biol 346:1121-1145.
9. Daggett V, Fersht AR (2003) The present view of the mechanism of protein folding. Nat Rev Mol Cell Biol 4: 497-502.
10. Karanicolas J, Brooks CL (2003) Improved Go-like models demonstrate the robustness of protein folding mechanisms towards non-native interactions. J Mol Biol 334:309-325.
11. Matysiak S, Clementi C (2004) Optimal combination of theory and experiment for the characterization of the protein folding landscape of S6: how far can a minimalist model go? J Mol Biol 343:235-248.
12. Das P, Matysiak S, Clementi C (2005) Balancing energy and entropy: a minimalist model for the characterization of protein folding landscapes. Proc Natl Acad Sci USA 102:10141-10146.
13. Kaya H, Chan HS (2002) Towards a consistent modeling of protein thermodynamic and kinetic cooperativity: how applicable is the transition state picture to folding and unfolding? J Mol Biol 315:899-909.
14. Kaya H, Chan HS (2004) Origins of chevron rollovers in non-two-state protein folding kinetics. Phys Rev Lett 90:A258104.
